# Autotrophic bacterial production of polyhydroxyalkanoates using carbon dioxide as a sustainable carbon source

**DOI:** 10.3389/fbioe.2025.1545438

**Published:** 2025-06-04

**Authors:** Ganesan Sathiyanarayanan, Sandra Esteves

**Affiliations:** Wales Centre of Excellence for Anaerobic Digestion, Sustainable Environment Research Centre, University of South Wales, Pontypridd, United Kingdom

**Keywords:** polyhydroxyalkanoates, carbon dioxide, CO_2_ fixation, autotrophs, cyanobacteria, photosynthetic bacteria, hydrogen-oxidizing bacteria, genetic engineering

## Abstract

The persistence of fossil fuel-based plastics poses significant environmental challenges, prompting increased research into biodegradable polyhydroxyalkanoate (PHA) polymers derived from cost-effective and sustainable resources. Different microorganisms can produce PHA amongst carbon dioxide (CO_2_)-assimilating autotrophic organisms, particularly noteworthy in carbon capture and utilization (CCU). Autotrophic bacteria have evolved to utilize either light (photoautotrophy) or inorganic chemicals (chemolithoautotrophy) to capture CO_2_, which powers their primary and secondary metabolic activities. This review explores the diversity of PHA-producing autotrophs, the metabolic pathways implicated in autotrophic PHA accumulation, and recent progress in photoautotrophs and chemolithoautotrophs regarding PHA synthesis using CO_2_. Additionally, microbial electrosynthesis for converting CO_2_ to PHA is also discussed. Genetic engineering strategies are also emphasized for the autotrophic synthesis of PHA. This review also addresses the challenges and prospects for sustainable PHA production using CO_2_.

## 1 Introduction

Polyhydroxyalkanoates (PHAs) are a diverse group of microbial polyesters synthesized intracellularly by various microorganisms including bacteria, archaea, cyanobacteria as carbon and energy storage compounds (Rehm, 2010). These polyesters are of significant interest due to their biodegradability, thermoplasticity, and potential as sustainable alternatives to petrochemical-based plastics (Reddy et al., 2003; Możejko-Ciesielska and Kiewisz, 2016; Pandey et al., 2022). PHAs are accumulated as discrete granules within the cytoplasm under imbalanced growth conditions and are mobilized by cells under nutrient-limiting scenarios (Behera et al., 2022).

PHAs are structurally classified based on the number of carbon atoms in their monomer units. The two main categories are short chain-length PHAs (SCL-PHAs), which consist of three to five carbon atoms, and medium chain-length PHAs (MCL-PHAs), comprising six to fourteen carbon atoms (Anjum et al., 2016). SCL-PHAs include well known types such as poly-3-hydroxybutyrate (PHB), poly-3-hydroxyvalerate (PHV), and their copolymeric product poly-3-hydroxybutyrate-co-3-hydroxyvalerate (PHB-co-PHV). MCL-PHAs include polymers like poly-3-hydroxy octanoate (PHO), poly-3-hydroxy hexanoate (PHHx), poly-3-hydroxy decanoate (PHD), poly-3-hydroxy dodecanoate (PHDD), poly-3-hydroxy heptanoate (PHH). To date, over 150 PHA monomeric types have been discovered, underscoring PHAs as the most structurally diverse group of natural polyesters (Muneer et al., 2020).

The biosynthesis of PHA is primarily triggered under conditions of nutrient imbalance typically, an excess carbon sources combined with limitations in essential nutrients such as nitrogen, magnesium, phosphorous, sulphur, and oxygen (Sudesh et al., 2000; Reddy et al., 2003; Passanha et al., 2013; Sathiyanarayanan et al., 2013a). Additionally, environmental stressors such as temperature fluctuations, high osmotic pressure, and extreme pH conditions can induce PHA synthesis (Passanha et al., 2014; Obruca et al., 2020). These environmental triggers exploited in both natural ecosystems and controlled fermentation processes to maximise PHA production yields.

From an industrial perspective, PHAs represent a promising class of biodegradable polymers synthesised from renewable sources including agricultural residues, municipal wastes, and industrial by-products (Jiang et al., 2016). Moreover, PHAs are completely biodegradable and highly biocompatible, making them ideal for various applications (Pandey et al., 2022). In the biomedical sector, PHAs are employed in drug delivery systems, scaffolds for tissue engineering, and resorbable sutures due to their favourable degradation kinetics and non-toxic breakdown products (Gregory et al., 2022). In packaging, PHAs are being increasingly adopted as green alternatives to single-use plastics, offering compostable options for containers (Park et al., 2024). In agriculture, PHA-based films are used in the development of controlled-release fertilizers and biodegradable plant pots (Amelia et al., 2019). Furthermore, PHAs are used in the production of sustainable consumer goods, such as disposable cutlery, shopping bags, and cosmetic containers, contributing significantly to the global initiative against plastic pollution (Pandey et al., 2022).

The commercialisation of PHAs faces several key challenges. Majorly, high production costs, driven by expensive substrates (Sathiyanarayanan et al., 2013b; Li and Wilkins, 2020; Choi et al., 2023) and complex bacterial cultivation processes (i.e., heterotrophic) (Sathiyanarayanan et al., 2013c; Możejko-Ciesielska and Kiewisz, 2016), make PHA less competitive than petrochemical-based plastics. Also, optimising yields and productivity on an industrial scale remains difficult despite advances in metabolic engineering and process optimisation (Akaraonye et al., 2010; Behera et al., 2022). Therefore, the commercial viability of large-scale industrial PHA production depends on developing efficient fermentation processes that use low-cost carbon sources (Choi et al., 2023). Numerous attempts have been made to synthesise PHAs using cost-effective substrates, such as industrial and agricultural wastes, as carbon sources (Li and Wilkins, 2020; Choi et al., 2023; Kedia et al., 2014; Kumi et al., 2016; Tao et al., 2016). However, these production processes often have a significant carbon footprint (Baioli et al., 2019).

The climate and energy plans aim to reduce greenhouse gas (GHG) emissions in Europe by at least 40% below 1990 levels by 2030, with an ambition to further decrease emissions by 80%–95% by 2050 (European commission, 2021). Carbon dioxide (CO_2_) is the primary GHG released through anthropogenic activities. Currently, CO_2_ is an abundant resource on Earth and can be utilised to produce carbon-based chemicals (Francisco et al., 2019). Carbon Capture and Utilisation (CCU) technologies employ CO_2_ as a raw material for the synthesis of fuels, polymers, and building materials through chemical reduction processes (Muthuraj and Mekonnen, 2018; Grignard et al., 2019; Francisco et al., 2019; Liu C. et al., 2015). The biocatalytic reduction processes including gas fermentation and microbial electrosynthesis are also kind of CCU technologies that enables the conversion of C_1_ gaseous feedstocks (e.g., CO, CO_2_, CH_4_, syngas, or biogas) into valuable products by means of microorganisms (Teixeira et al., 2018). The production of bioplastics such as PHAs from C_1_ gas feedstocks represents a particularly compelling application of CCU. This technology has already achieved a semi-commercial scale, exemplified by microbial production of PHAs from CH_4_ (Newlight Technologies, 2024)

**FIGURE 1:**
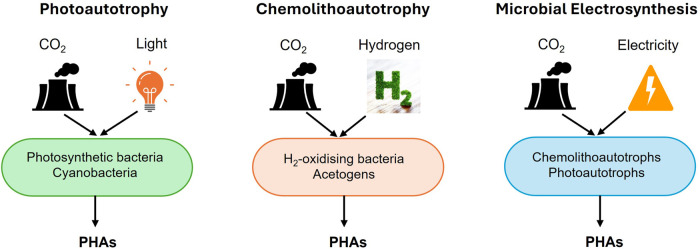
Production of PHAs from CO_2_ via different autotrophic metabolisms. Autotrophic microorganisms can fix and convert CO_2_ into cellular biomass and PHAs. This process requires an external energy source to drive CO_2_ reduction. Depending on the type of autotrophy, energy can be derived from light (photoautotrophy), inorganic electron donors like hydrogen (chemolithoautotrophy), or from an electrode at a poised potential (microbial electrosynthesis). In all cases, CO_2_ serves as the sole carbon source for both growth and PHA biosynthesis.

Using C_1_ gases as feedstocks is likely to result in the production of PHA with a low carbon footprint (Khosravi-Darani et al., 2013a; Azim et al., 2020). This approach offers the added benefits of consistent feed quality and reduced contamination risks compared to substrates derived from organic wastes (Ma et al., 2024). Additionally, certain bacteria, known as autotrophs, have the ability to reduce or fix CO_2_ into bio-based products, including PHA (Srisawat et al., 2022b). As the most oxidized C_1_ feedstock, CO_2_ requires a high energy input to be converted into more reduced chemical products like PHA. This energy can be supplied through light, as utilized by photosynthetic microorganisms (photoautotrophy) (Liebergesell et al., 1991; Carpine et al., 2020) or inorganic compounds such as hydrogen (H_2_) (chemolithoautotrophy) (Liebergesell et al., 1991) or via more efficient sources of reducing power, such as bio-electrocatalysis (microbial electrosynthesis) (Pepè Sciarria et al., 2018; Banu et al., 2019). Autotrophic metabolisms discussed in this review are illustrated in Figure 1.

Autotrophic PHA synthesis from CO_2_ is known for its sustainable carbon utilization and energy efficiency compared to heterotrophic synthesis, wherein PHAs are produced from organic substrates. Despite extensive efforts to synthesize PHAs using both heterotrophic and autotrophic microorganisms, the application of autotrophic systems, particularly those relying on CO_2_ as a carbon source, remains underexplored. This is primarily due to the persistent challenges of achieving efficient production yields under autotrophic conditions. This review briefly discusses the biodiversity of PHA synthesizing autotrophs and autotropic PHA synthesis metabolisms. In addition, photoautotrophs, chemolithoautotrophs, and microbial electrosynthesis are highlighted for the autotrophic synthesis of PHA using CO_2_ as a substrate. Finally, genetic engineering strategies in developing CO_2_-fixing autotrophic microbial cell factories for PHA synthesis are also elucidated.

## 2 Biodiversity of PHA-producing heterotrophs and autotrophs

The earliest discovery of bacterial PHAs, specifically PHB, was documented in 1926 from the *Priestia megaterium*, previously classified as *Bacillus megaterium* (Lemoigne, 1926). Since then, various heterotrophic bacterial phyla, including Proteobacteria (α, β, γ, and δ), Firmicutes (Bacilli and Clostridia), Bacteroidetes, Actinobacteria, Deinococcus-Thermus, and Cyanobacteria, have been documented for PHA synthesis (Li and Wilkins, 2020; Behera et al., 2022; Saravanan et al., 2022). Currently, more than 92 bacterial genera are known for PHA synthesis. Most of them were isolated and screened from diverse environmental niches such as soil, freshwater, marine water, polar environments, and hydrothermal vents (Liu et al., 2024). Notable PHA-producing genera are *Aeromonas*, *Alcaligenes*, *Azotobacter*, *Burkholderia*, *Cupriavidus*, *Chelatococcus*, *Comamonas*, *Corynebacterium, Enterobacter*, *Methylobacterium*, *Pseudomonas*, *Rhodobacter*, *Rhodopseudomonas*, *Sinorhizobium*, and *Thermus* (Reddy et al., 2003; Li and Wilkins, 2020). SCL-PHAs are synthesized heterotrophically by numerous species, including *Cupriavidus necator, Burkholderia cepacia*, and *Alcaligenes latus*. Simultaneously, MCL-PHAs can be synthesized by fluorescent *Pseudomonas* species, including *P. putida*, *P. oleovorans*, and *P. corrugate* (Reddy et al., 2003; Behera et al., 2022). Some bacteria, including *Aeromonas hydrophila* (Możejko-Ciesielska et al., 2019) and *Thiococcus pfennigii* (Liebergesell et al., 2000), synthesize both SCL- and MCL-PHAs copolymers. In the Archaea domain, only haloarchaeal genera are known to produce PHAs, mainly *Haloferax, Haloarcula, Halorubrum, Halobacterium, Haloterrigena, Halococcus, Haloquadratum, Natronobacterium, Natrialba,* and *Natronococcus* (Koller and Rittmann, 2022)*.* Among haloarchaea, *Haloferax mediterranei* is particularly noteworthy for its capability to synthesize substantial quantities of PHB-co-PHV copolymer (Poli et al., 2011; Koller and Rittmann, 2022). In recent decades, wild-type bacterial strains (i.e., *Escherichia coli*, *P. putida,* and *C. necator*) were also genetically/metabolically engineered for the commercial production of PHAs under heterotrophic cultivation (Li and Wilkins, 2020; Saravanan et al., 2022; Wang et al., 2023).

**FIGURE 2:**
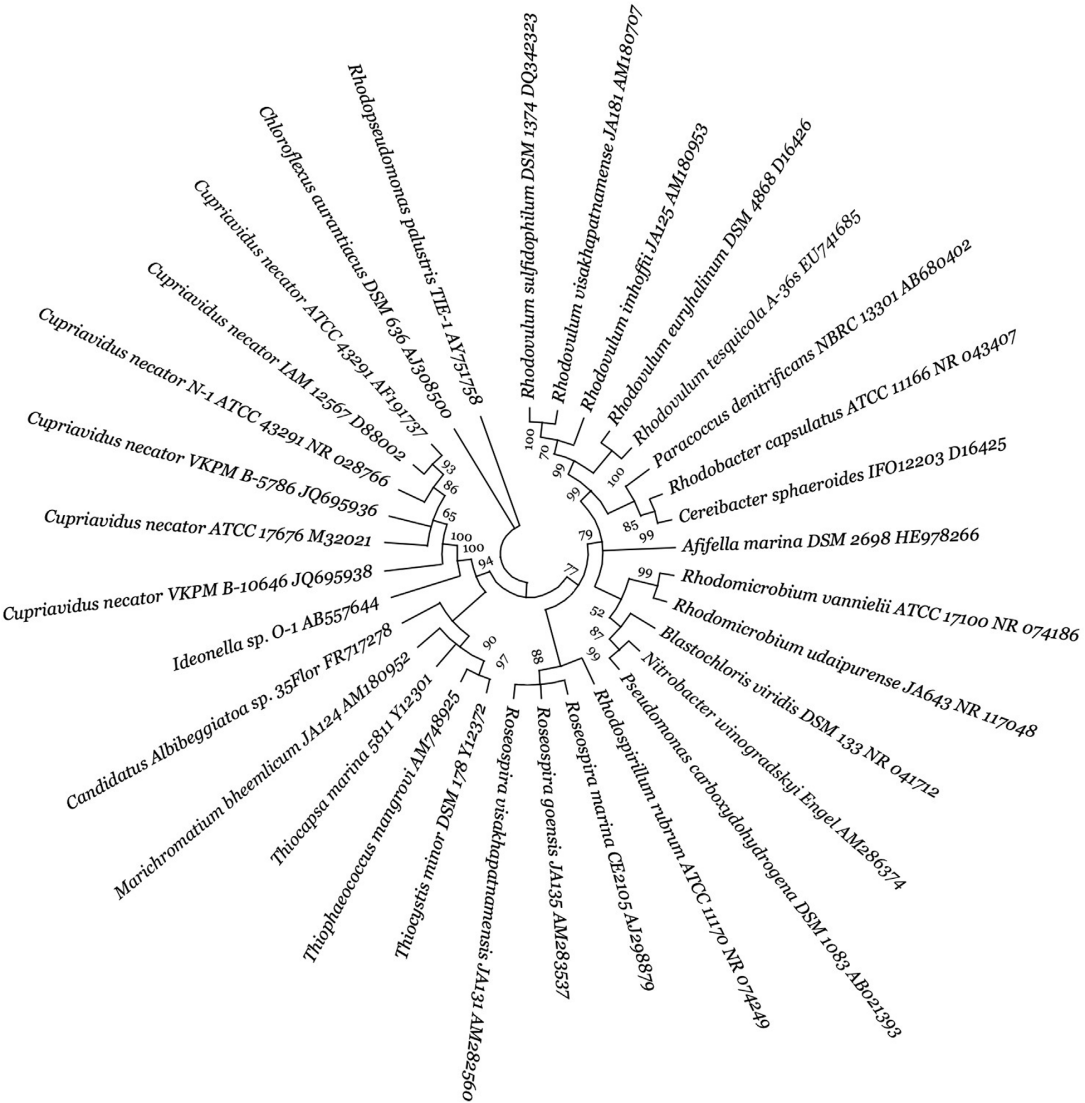
Diversity of autotrophic PHA-producing photosynthetic and chemolithotrophic bacteria. The figure presents a molecular phylogenetic analysis of 16S rRNA gene sequences using the neighbour-joining method for selected bacteria. For detailed methodology, refer to Supplementary data. The proportion of trees (≥70%) where the accompanying taxa clustered is indicated next to the branch nodes, based on 1,000 iterations. The strain number and GenBank accession numbers are properly designated for each microorganism.

**FIGURE 3:**
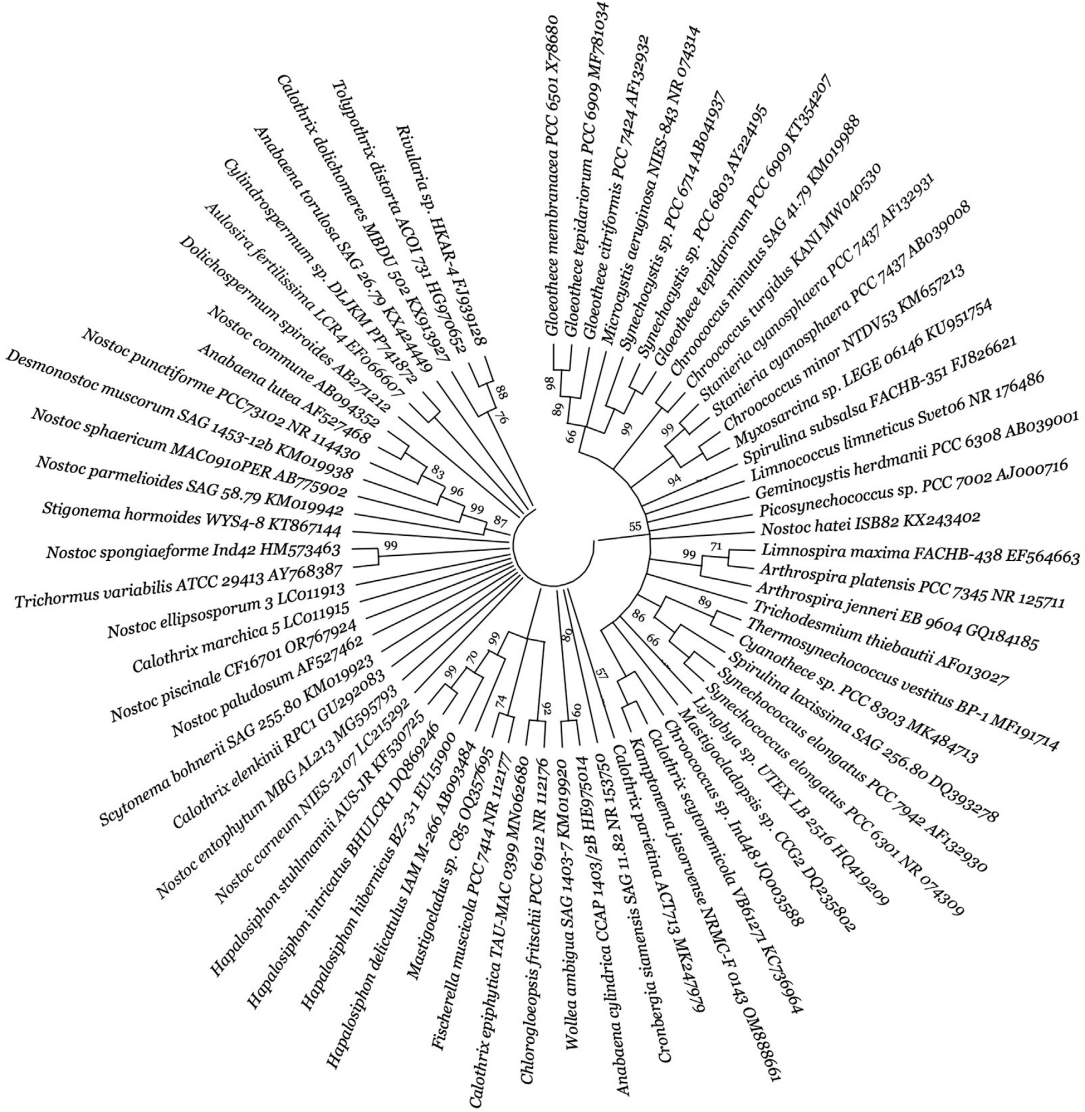
Diversity of autotrophic PHA-producing cyanobacteria. The figure presents a molecular phylogenetic analysis of 16S rRNA gene sequences using the neighbour-joining method for selected bacteria. For detailed methodology, refer to Supplementary data. The proportion of trees (≥70%) where the accompanying taxa clustered is indicated next to the branch nodes, based on 1,000 iterations. The strain number and GenBank accession numbers are properly designated for each microorganism.

This review surveyed the vast diversity of PHA-synthesizing CO_2_-fixing autotrophic microorganisms (Figures 2, 3), and their phylogenetic tree generation methods have been emphasized in the supplementary material as supplementary data. Microbial biodiversity information is highly crucial for understanding PHA biosynthetic pathways and developing efficient PHA-producing autotrophic microbial cell factories. In the realm of CO_2_-fixing autotrophs, both photoautotrophic and chemolithoautotrophic group of microorganisms are prominent producers of PHAs (Srisawat et al., 2022b). Photoautotrophs can be classified into oxygenic (e.g., cyanobacteria) and anoxygenic bacteria (e.g., purple non-sulphur bacteria, PNSB). Within cyanobacteria, *Anabaena* sp. PCC 7120, *Synechococcus elongatus* PCC 7942, *Synechococcus* sp. PCC 7002 and *Synechocystis* sp. PCC 6803, and regarded as model organisms for photoautotrophic PHA production (Troschl et al., 2017a; Srisawat et al., 2022b; Ray et al., 2023). Anoxygenic PNSB, for example, freshwater *Rhodobacter sphaeroides* (Liebergesell et al., 1991; Schmid et al., 2021; Li et al., 2023), *Rhodopseudomonas palustris* (Ranaivoarisoa et al., 2019; Li et al., 2022), *Rhodospirillum rubrum* (Liebergesell et al., 1991; Revelles et al., 2016), *Rhodobacter capsulatus* (Liebergesell et al., 1991), *Rhodomicrobium vannielii* (Conners et al., 2023) and marine water *Rhodovulum sulfidophilum* (Higuchi-Takeuchi et al., 2016a; 2016b), have shown significant potential for PHA production, contributing to synthesizing biopolymers like PHB and other value-added chemicals. Chemolithoautotrophs, in contrast, utilize inorganic energy sources (i.e., H_2_, Fe^2+^, NH_3_, H_2_S) instead of light. Notable examples are *C. necator* (Tanaka et al., 1995; Volova et al., 2013) and *Ideonella* sp. O-1 (Tanaka et al., 2011) can grow on a gas mixture of CO_2_, H_2_, and O_2_, producing PHB very effectively. In addition to the above two groups, there are acetogens can fix CO_2_ to produce various bioproducts (Debabov, 2021; Flaiz and Sousa, 2024). Key acetogenic mixotrophs capable of fixing CO_2_ to produce biomolecules include *Acetobacterium woodii*, *Butyribacterium methylotrophicum, Blautia producta, Clostridium aceticum*, *C. autoethanogenum*, *Clostridium ljungdahlii*, and *C. carboxidivorans* (Salehizadeh et al., 2020). Acetogens are unable to synthesise PHAs naturally due to absence of PHA biosynthetic genes. However, genetically engineered *C. coskatii* has been shown to synthesize PHA via autotrophic CO_2_ reduction (Flüchter et al., 2019).

## 3 Autotrophic PHA synthesis metabolisms

Extensive genomic and metabolic research has significantly enhanced our understanding of PHA biosynthesis and degradation. Understanding heterotrophic PHA synthesis is essential before exploring autotrophic pathways because it provides the foundational knowledge of the core biosynthetic enzymes, regulatory mechanisms, and metabolic fluxes (Chen and Jiang, 2018). This baseline is crucial, as autotrophic systems often rely on the heterologous expression of these genes (Kourmentza et al., 2017). Moreover, it allows for comparative evaluation of yields, substrate utilization, and process efficiency in engineered autotrophic platforms. The heterotrophic bacterial synthesis of PHA requires two primary stages: the generation of hydroxyacyl-CoA and its subsequent polymerization into PHA (Sudesh et al., 2000; Reddy et al., 2003). In the initial stage, three principal metabolic pathways facilitate PHA production: acetoacetyl-CoA generation, *de novo* lipogenesis, and β-oxidation (Haddadi et al., 2019). Acyl-CoA and acetyl-CoA are predominant intermediates across these metabolic pathways (Muneer et al., 2020) and regulate PHA production (Luengo et al., 2003). Depending on carbon sources, heterotrophic bacteria can synthesize PHA using different metabolic pathways. The acetoacetyl-CoA generation and *de novo* lipogenesis pathways execute the PHA synthesis when the medium is amended with a sugar substrate. In contrast, the fatty acid β-oxidation pathway significantly contributes to PHA production when fatty acids are the primary carbon source. All these pathways lead to the polymerization reaction catalysed by the enzyme PHA synthase.

**FIGURE 4:**
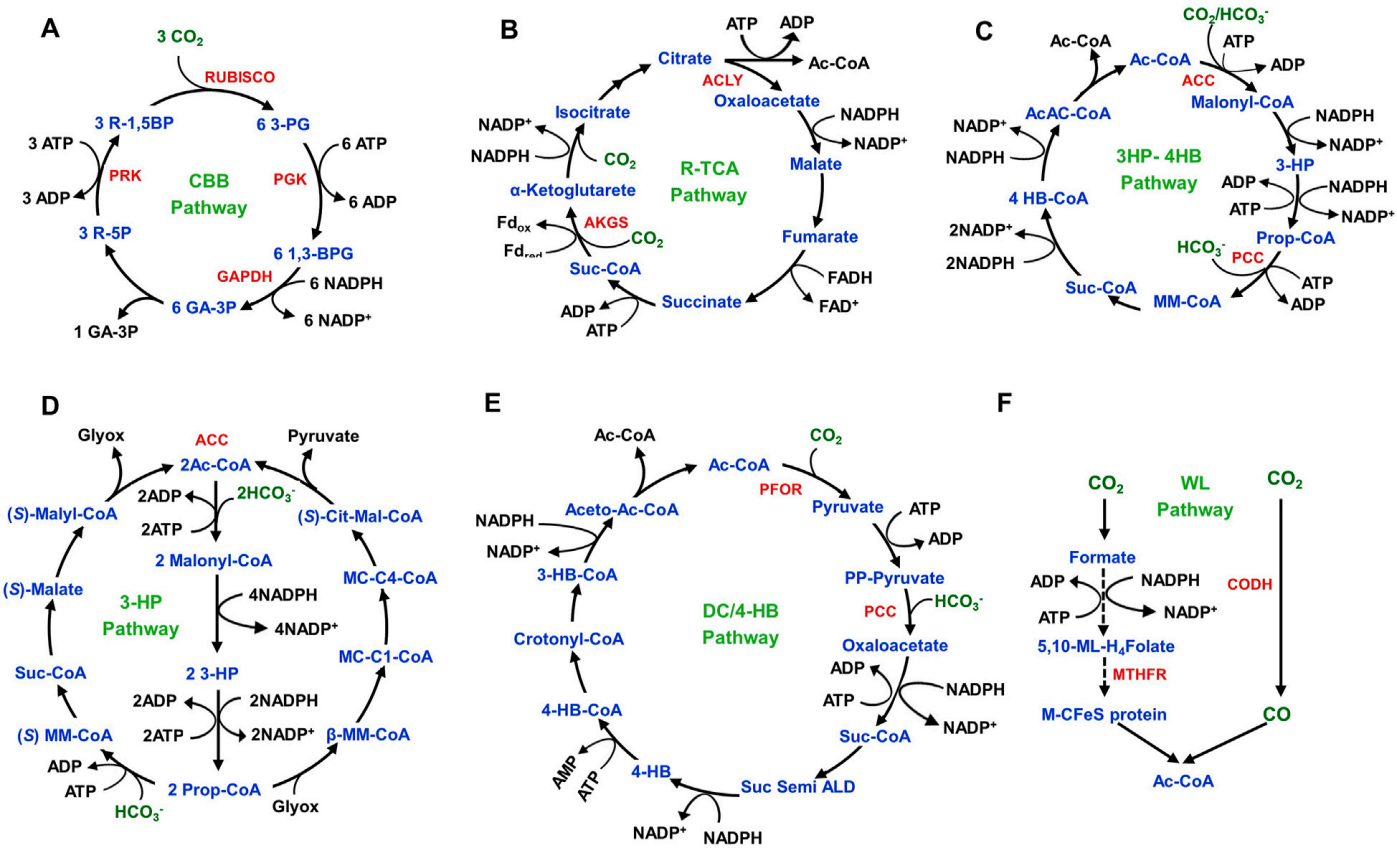
CO_2_-fixation metabolic pathways in autotrophic microorganisms. **(A)** Calvin‒Benson‒Bassham (CBB) pathway in cyanobacteria, algae, and proteobacteria. **(B)** Reductive TCA pathway in proteobacteria, green sulphur bacteria and Aquificae bacteria **(C)** 3-Hydroxypropionate/4-hydroxybutyrate (3HP-4HB) pathway in aerobic crenarchaeota. **(D)** 3-Hydroxypropionate (3-HP) pathway in green non-sulphur bacteria. **(E)** Dicarboxylate/4-hydroxybutyrate (DC/4-HB) pathway in anaerobic crenarchaeota. **(F)** Wood–Ljungdahl (WL) pathway in proteobacteria, spirochetes, planctomycetes and Euruarchaeota. The solid line represents a single reaction. The dashed line represents multiple reactions. The abbreviations of the metabolites (blue colour) are as follows: 3-PG, 3-Phosphoglycerate; 1,3-BPG: 1,3-Bisphophoglycerate; GA-3P: Glyceraldehyde-3-Phosphate; R-5P: Ribulose-5-Phosphate; R-1,5BP: Ribulose-1,5-Bisphosphate; Ac-CoA: Acetyl-CoA; 3-HP, 3-Hydroxypropionic acid; Prop-CoA, Propionyl-CoA; MM-CoA: Methylmalonyl-CoA; suc-CoA, Succinyl-CoA; 4 HB-CoA, 4-hydroxybutyryl-CoA; AcAc-CoA: Acetoacetyl-CoA; *(S)*-Cit-Mal-CoA: Citramalyl-CoA; MC-C4-CoA, Mesancolyl-C_4_-CoA; MC-C1-CoA, Mesancolyl-C_1_-CoA; β-MM-CoA: β-Methylmalyl-CoA; PP-Pyruvate, Phosphoenolpyruvate; Suc Semi ALD, Succinate Semialdehyde; 4-HB, 4-Hydroxybutyrate; 3-HB-CoA, 3-Hydroxybutyryl-CoA; Aceto-Ac-CoA, Acetoacetyl-CoA; 5,10-ML-H_4_Folate, 5,10-Methenyl-H_4_Folate; M-CFeS protein, Methyl-corrinoid iron-sulphur protein. The abbreviations of the metabolic enzymes (red colour) are as follows: RuBisCO, ribulose-1,5-bisphosphate carboxylase/oxygenase; PGK, phosphoglycerate kinase; GAPDH, glyceraldehyde-3-phosphate dehydrogenase; PRK, phosphoribulokinase; ACLY, ATP-citrate lyase; AKGS, α-ketoglutarate synthase; ACC, acetyl-CoA carboxylase; PCC, pyruvate carboxylase; PFOR, pyruvate ferredoxin oxidoreductase; CODH, carbon monoxide dehydrogenase; MTHFR, methylenetetrahydrofolate reductase.

Autotrophic PHA synthesis mainly relies on CO_2_ fixation in microorganisms. Until now, six distinct pathways have been recognized for microbial CO_2_ fixation such as reductive pentose phosphate cycle/Calvin-Benson-Bassham (CBB) pathway, Wood-Ljungdahl (WL) pathway, reductive tricarboxylic acid (R-TCA) pathway, 3-Hydroxypropionate pathway (3-HP/malyl-CoA), 3-Hydroxypropionate/4-Hydroxybutyrate (3HP-4HB) pathway, and dicarboxylate/4-Hydroxybutyrate (DC/4-HB) pathway (Figure 4). Mostly, cultivation conditions determine CO_2_ fixation pathways in microorganisms. Aerobic conditions enable 3HP-4HB, 3-HP/malyl-CoA, and CBB pathways, but anaerobic nature activates WL, DC/4HB, and R-TCA pathways. The other four pathways share similarities except for the WL and the DC/4HB pathways. The WL pathway is a linear carbon fixation mechanism that converts CO_2_ into acetyl-CoA and is predominantly found in acetogenic bacteria.

Photoautotrophs, comprising both oxygenic and anoxygenic types, utilize light energy to produce ATP (Claassens et al., 2016). Oxygenic photoautotrophs, such as cyanobacteria, produce reducing power and create a proton gradient essential for ATP synthesis through the action of photosystem I and II complexes, which split water molecules and release oxygen. The light-dependent electron transport chain supplies the energy and reduces equivalents required for CO_2_ fixation via the CBB pathway, enabling the autotrophic synthesis of essential cellular components and various metabolites (Kanno et al., 2017). Anoxygenic photolithoautotrophs, such as PNSB, possess a single photosystem incapable of splitting water. Instead, they rely on organic and inorganic compounds such as H_2_ and sulphur compounds as electron donors to produce reducing power for light-driven CO_2_ fixation through the CBB pathway (Inui et al., 1998). PNSB also has a highly flexible metabolism, which allows them to thrive under aerobic and anaerobic conditions and exhibit both autotropic and heterotrophic growth. Their competence to adapt to extreme environments makes them model organisms for producing PHAs. The facultative chemolithoautotrophic bacterium *C. necator* can also fix CO_2_ via the CBB pathway while utilizing H_2_ as its exclusive energy source, even in the presence of O_2_ (Morlino et al., 2023). Therefore, the CBB pathway is predominantly present in most autotrophs, including cyanobacteria, algae, photoautotrophic, and chemolithoautotrophic bacteria. Thus, further exploring the CBB pathway is essential to understand autotrophic PHA synthesis.

**FIGURE 5:**
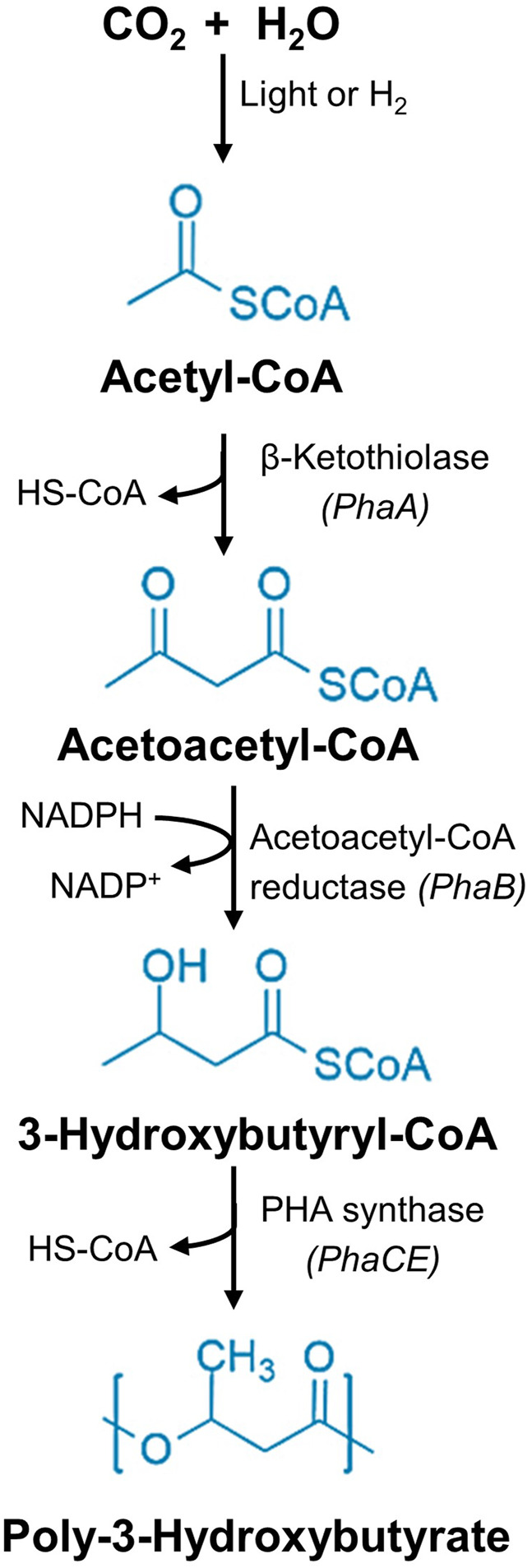
PHB synthesis route. Common metabolic pathway of the photoautotrophic (Cyanobacteria) and chemolithoautotrophic bacteria (*C. necator*) for the biosynthesis of PHB utilising CO_2_ as the primary carbon source.

In cyanobacteria, de nova lipogenesis (DNL) and nitrogen utilization are suggested mechanisms for producing PHAs (Troschl et al., 2017a; Carpine et al., 2020). Cyanobacteria can perform oxygenic photosynthesis through the CBB pathway, which generates ATP and NADPH to energise cellular activities. The cyanobacterial CBB pathway comprises three stages: the carboxylation of Ribulose-1,5-bisphosphate (RuBP), reduction of 3-phosphoglycerate (PGA), and regeneration of RuBP. In the carboxylation phase, three molecules of CO_2_ are fixed with six molecules of RuBP, forming six molecules of 3-phosphoglycerate (3-PGA). During the subsequent reduction stage, ATP and NADPH are utilized to transform 3-PGA into triose phosphate and dihydroxyacetone phosphate (DHAP). Finally, in the regeneration stage, five molecules of 3-PGA are used to regenerate three molecules of RuBP. Key enzymes involved in the CBB pathway are ribulose-1,5-bisphosphate carboxylase/oxygenase (RuBisCO), phosphoribulokinase (PrkA), and sedoheptulose bisphosphatase (SBPase) (Kumar M. et al., 2018). Cyanobacteria can assimilate CO_2_ and bicarbonate (HCO_3_^−^) through carbon dioxide-concentrating mechanisms (CCMs). This carbon assimilation involves five different transport systems. *Bic*A*, Sbt*A, and BCT1 enzymes facilitate the HCO_3_^−^ transport, while NDH-I_3_ and NDH-I_4_ enable CO_2_ assimilation. The transport of CO_2_ takes place via CO_2_ transporters situated in the plasma membrane, while HCO_3_^−^ transporters accelerate the translocation of intracellular HCO_3_^−^ across the plasma membrane. Additionally, periplasmic carbonic anhydrase induces the transformation of HCO_3_^−^ to CO_2_ (Durall and Lindblad, 2015). Depending on nutrient availability, the excess 3-PGA is channelled into synthesizing cellular materials. In nutrient-limited conditions, 3-PGA is diverted to synthesize PHAs. This process involves several key enzymes: β-keto thiolase (*phaA*), which catalyses the conversion of acetyl-CoA to acetoacetyl-CoA; acetoacetyl-CoA reductase (*phaB*), which condenses acetoacetyl-CoA to 3-hydroxy butyryl-CoA; and PHA synthase (*phaC*), which polymerizes 3-hydroxy butyryl-CoA into PHB (Gaspar et al., 2000). A simplified PHB synthesis metabolic route from the CO_2_ is illustrated in Figure 5.

Importantly, PHA synthase can integrate various hydroxy acid monomers into PHAs. This enzyme is categorized into four distinct classes: Class I (*PhaC*) found in *C. necator*, Class II (*PhaC*) present in *P. oleovorans*, Class III (comprising *PhaC* and *PhaE* subunits) found in *Allochromatium vinosum* and *Thiocapsa pfennigii*, and Class IV (consisting of *PhaC* and *PhaR* subunits) identified in *B. megaterium* (Luengo et al., 2003; Behera et al., 2022; Gregory et al., 2022). Cyanobacteria exclusively contain Class III PHA synthase (Carpine et al., 2020). Their genetic organization differs from other bacteria; in contrast to the single operon containing all four genes found in different bacterial species, cyanobacteria have two distinct operons. In the first operon, the *phaA* and *phaB* genes are co-expressed, while the *phaE* and *phaC* genes are in the second operon (Troschl et al., 2017a; Carpine et al., 2020).

In *C. necator*, the CBB pathway consists of 11 stages, where the RuBisCO enzyme primarily executes the CO_2_ fixation (Li et al., 2020). All enzymes necessary for CO_2_ fixation are encoded within the *cbb* operon, which appears in two copies in *C. necator* (Panich et al., 2021). Both copies of this operon are crucial for autotrophic growth. Additionally, the CBB pathway in *C. necator* demands significant energy input, requiring a net total of 7 mol of ATP to convert 3 mol of CO_2_ into 1 mol of pyruvate (Panich et al., 2021). Furthermore, RuBisCO in *C. necator* operates relatively slowly as a carboxylase and exhibits oxygenase activity, producing a toxic molecule called 2-phosphoglycolate (2-PG). This compound is not essentially required for further CBB mechanisms. Hence, it must be eliminated through a process known as 'phosphoglycolate salvage’ (Li et al., 2020; Panich et al., 2021). Most of the photoautotrophs execute the CCMs to balance the weak performance of the RuBisCO, whereas *C. necator* lacks typical CCM features. Instead, it captures CO_2_ using four metalloproteins (carbonic anhydrase), which help to accumulate adequate HCO_3_^−^ in the cytoplasm, which activates the RuBisCO enzyme to perform the CO_2_ fixation. *C. necator* can also produce an alternate RuBisCO-like enzyme with a high affinity to CO_2_ (465 nmol/min/mg) and a median rate of ∼2.5 s^-1^ (Li et al., 2020; Panich et al., 2021). All these enzyme systems help the *C. necator* to fix CO_2_ effectively to synthesize PHA.

## 4 Photoautotrophic synthesis of PHA from CO_2_

Photoautotrophs are organisms that perform photosynthesis. In the natural environment, they capture the light energy from sunlight to transform CO_2_ and water into organic compounds, which are then utilized for cellular processes such as biosynthesis and respiration. Photoautotrophic microorganisms include anoxygenic photosynthetic bacteria, cyanobacteria, and microalgae, and all these microorganisms are known to accumulate PHA (Liebergesell et al., 1991; Troschl et al., 2017a; Costa et al., 2019). In this review, particular emphasis has been given to procaryotic microorganisms such as anoxygenic photosynthetic bacteria and cyanobacteria as the autotrophic microbial cell factories for PHA production. Hence, eucaryotic microalgae are excluded.

### 4.1 Anoxygenic photosynthetic bacteria

Anoxygenic photosynthetic bacteria are classified into four clusters depending on their pigments and electron donors: green sulphur, green non-sulphur, purple sulphur, and purple non-sulphur bacteria. These bacteria obtain electrons from organic compounds, sulphur, and H_2_. Most anoxygenic phototrophs can function as either photoautotrophs or photoheterotrophs in the presence of light. At the same time, some species can grow as chemoheterotrophs in the absence of light. This chemoheterotrophic nature has facilitated the exploration of these bacteria for various applications, including industrial wastewater purification and H_2_ production. A list of autotrophic PHA production studies on anoxygenic photosynthetic bacteria is listed in Table 1.

**TABLE 1:** Photoautotrophic synthesis of PHA from photosynthetic bacteria.

Photosynthetic bacteria	Carbon source	PHA content in DCW (%)	PHA titre (mg/L)	Limiting factor	PHA composition	Bioreactor	References
Purple non-sulfur bacteria							
*Afifella marina* DSM 2698	0.1% NaHCO_3_	<0.1–4%	-	N	PHB	Erlenmeyer Flask	Higuchi-Takeuchi et al. (2016b)
*Rhodovulum euryhalinum* DSM 4868	0.1% NaHCO_3_	<0.1–4%	-	N	PHB	Erlenmeyer Flask	Higuchi-Takeuchi et al. (2016b)
*Rhodovulum imhoffii* JCM 13589	0.1% NaHCO_3_	<0.1–4%	-	N	PHB	Erlenmeyer Flask	Higuchi-Takeuchi et al. (2016b)
*Rhodovulum sulfidophilum* ATCC 35886	0.1% NaHCO_3_	<0.1–4%	-	N	PHB	Erlenmeyer Flask	Higuchi-Takeuchi et al. (2016b)
*R. sulfidophilum* ATCC 35886	20 mM NaHCO_3_ ^a^	-	21.23	-	PHA^b^	Tube culture	Srisawat et al. (2022a)
*R. sulfidophilum* ATCC 35886	20 mM NaHCO_3_ ^c^	-	11.28	-	PHA^b^	Tube culture	Srisawat et al. (2022a)
*Rhodospirillum rubrum* ATCC 11170	Syngas (40% H_2,_ 10% CO_2._ 40% CO, 10% N_2_) + 10 mM Acetate	28%	-	N	PHB	-	Revelles et al. (2016)
*R. rubrum* ATCC 11170	Syngas (40% H_2,_ 10% CO_2._ 40% CO, 10% N_2_) + 10 Mm Acetate	20%	-	-	PHB	Bottles	Revelles et al. (2017)
*R. rubrum* ATCC 11170	Syngas (37% H_2,_ 6% CO_2._ 27% CO, 26% N_2._ 4% CH_4_) + 10 Mm Acetate	16%	-	-	PHB	Bottles	Revelles et al. (2017)
*R. rubrum* ATCC 11170	Syngas (25% CO, 25% H_2_, 5% CO_2_, 45% N_2_) + Acetate	30%	-	-	PHB	Bottles	Karmann et al. (2019)
*R. rubrum* ATCC 11170	Syngas (15% CO, 85% N_2_) + 3 mM Acetate	8%	-	-	PHB	Erlenmeyer Flask	Mongili and Fino (2021)
*Rhodovulum tesquicola* ATCC BAA1573	0.1% NaHCO_3_	<0.1–4%	-	N	PHB	Erlenmeyer Flask	Higuchi-Takeuchi et al. (2016b)
*Rhodovulum visakhapatnamense* JCM 13531	0.1% NaHCO_3_	<0.1–4%	-	N	PHB	Erlenmeyer Flask	Higuchi-Takeuchi et al. (2016b)
*Roseospira marina* ATCC BAA 447	0.1% NaHCO_3_	<0.1–4%	-	N	PHB	Erlenmeyer Flask	Higuchi-Takeuchi et al. (2016b)
*Roseospira goensis* JCM 14191	0.1% NaHCO_3_	<0.1–4%	-	N	PHB	Erlenmeyer Flask	Higuchi-Takeuchi et al. (2016b)
*Roseospira visakhapatnamensis* ATCC BAA 1365	0.1% NaHCO_3_	<0.1–4%	-	N	PHB	Erlenmeyer Flask	Higuchi-Takeuchi et al. (2016b)
Purple sulfur bacteria							
*Thiocystis minor* (Chromatium minus)	CO_2_	-	-	-	PHB	-	Esteve et al. (1990)
*Thiohalocapsa marina* DSM 5653	0.1% NaHCO_3_	<0.1–4%	-	N	PHB	Erlenmeyer Flask	Higuchi-Takeuchi et al. (2016b)
*Thiophaeococcus mangrove* JCM 14889	0.1% NaHCO_3_	<0.1–4%	-	N	PHB	Erlenmeyer Flask	Higuchi-Takeuchi et al. (2016b)
*Marichromatium bheemlicum* JCM 13911	0.1% NaHCO_3_	<0.1–4%	-	N	PHB	Erlenmeyer Flask	Higuchi-Takeuchi et al. (2016b)
Green sulfur bacteria							
*Chloroflexus aurantiacus* OK-70fL (DSM 636)	H_2_:CO_2_ (80:20)	-	-	-	PHV, PHB-co-PHV	CSTR	van der Meer et al. (2001)

^a^ Culture medium was amended with anionic nano gel M55T43 (1.0 mg/mL).

^b^ PHA, monomer details not known.

^c^ Culture medium was amended with anionic nano gel A55T43 (1.0 mg/mL).

Abbreviations: N, Nitrogen (inorganic nitrogen source); CSTR, continuous stir tank reactor; NaHCO_3,_ sodium bicarbonate.

*R. rubrum* has been extensively studied for its competence in transforming syngas (a gas mixture of CO_2_, H_2_, CO, N_2_) into PHAs under photoautotrophic anaerobic conditions (Do et al., 2007; Revelles et al., 2016). *R. rubrum* can assimilate CO effectively as a sole carbon and energy source, wherein CO exposure induces a set of enzymes such as carbon monoxide dehydrogenase (CODH) and CO-tolerant hydrogenase, which further catalyse the oxidative conversion of CO to CO_2_ and H_2_, respectively. The resulting CO_2_ can be fixed by the CBB pathway for biomass production and subsequent PHA synthesis (Do et al., 2007). Moreover, when the syngas fermentation media was amended with acetate as an additional substrate, *R. rubrum* was shown to produce PHH up to 20% and 28% of its dry biomass under photoheterotrophic (light) and chemoheterotrophic (dark) settings, respectively (Revelles et al., 2016). In addition, *R. rubrum* had synthesized up to 16% of PHB from syngas derived from municipal solid waste when the process was amended with 10 mM acetate as a co-substrate (Revelles et al., 2017). In another study, diluted syngas and acetate combination also enhanced the PHB synthesis by up to 30% with a titre of 1.6 g/L under carbon and phosphorus limitation (Karmann et al., 2019).

Recently, a versatile nitrogen-fixing PNSB genus *Rhodomicrobium* was also identified as a PHA producer. *Rhodomicrobium vannielii* and *Rhodomicrobium udaipurense* have been shown to produce PHA under photoautotrophic (CO_2_) and photoheterotrophic (sodium butyrate) cultivation with either NH_4_Cl or N_2_ gas as nitrogen sources (Conners et al., 2024). During photoautotrophic cultivation with CO_2_, two different electron donors such as H_2_ (photohydrogenotrophy) and Fe^2+^ (photoferrotrophy), were used as an energy source. Photoferrotrophic growth resulted in a higher PHA synthesis in both species (4.64%–47.03% cdw_prot_) than photohydrogenotrophic growth (1.10%–6.19% cdw_prot_), where NH_4_Cl as a nitrogen source. N_2_-fixation promotes the PHA synthesis in photoheterotrophic growth but inhibits during the photoautotrophic condition in both species (Conners et al., 2024). A similar set of experiments was conducted on *R. palustris* TIE-1 (Ranaivoarisoa et al., 2019), where NH_4_Cl as a nitrogen source has produced higher PHB (7.23%, PHB carbon yield) in photo hydrogenotrophic growth than photoferrotrophic growth (5.77%, PHB carbon yield), whereas N_2_ fixing condition had shown a lower PHB yield (<3%, PHB carbon yield) in both photo hydrogenotrophic and photoferrotrophic growth under photoautotrophic condition (Ranaivoarisoa et al., 2019). In *Rhodomicrobium* and *Rhodopseudomonas*, N_2_-fixation does not effectively support the PHB synthesis under photoautotrophic CO_2_ reduction. Further, extensive studies may open new avenues for developing *Rhodomicrobium* and *Rhodopseudomonas* species as promising photoautotrophic platforms for PHA production.

Marine phototrophic bacteria are also considered excellent model organisms for the sustainable production of various products. They offer several benefits, including metabolic adaptability and tolerance to high salinity, which can help to develop low-cost, non-axenic fermentation processes (Higuchi-Takeuchi and Numata, 2019). Marine purple sulphur and PNSB have been explored for the synthesis of PHA under photoautotrophic conditions, where 1% sodium bicarbonate (NaHCO_3_, an inorganic source of CO_2_) is supplemented as a sole carbon source (Higuchi-Takeuchi et al., 2016a; 2016b). Among the species tested, very few PNSB (i.e., *R. sulfidophilum, R. imhoffii, R. euryhalinum,* and *R. visakhapatnamense*) were only able to synthesize PHA (up to <5%) under nitrogen-limited photoautotrophic conditions. Their biomass production was also lower than the photoheterotrophic condition (Higuchi-Takeuchi et al., 2016b). It was assumed that the low PHA production was due to the fluctuations in the cellular redox state and lower concentrations of NaHCO_3_ in the growth medium.

Photoautotrophic PHA synthesis in PNSB remains challenging since photoheterotrophic carbon assimilation pathways are less complex than photosynthetic carbon-fixation pathways. To overcome these hurdles, engineered nano-gel particles have been recently suggested to enhance the assimilation of NaHCO_3_ by *R. sulfidophilum* for the photoautotrophic synthesis of PHA (Srisawat et al., 2022a). Extensive screening of engineered anionic nano gel particles against the *R. sulfidophilum* biomass and PHA synthesis has increased up to 157-fold than control conditions without gel particles. Effective assimilation and subsequent incorporation of HCO_3_^−^ in the autotrophic PHA synthesis confirmed by ^13^C tracing with gas chromatography-mass spectral analysis (Srisawat et al., 2022a). Therefore, engineered nanogel applications in different species of photosynthetic bacteria may expand our knowledge and efficiency of autotrophic PHA synthesis. Another interesting autotrophic PHA synthesis was identified in the green sulphur bacterium *Chloroflexus aurantiacus* while performing ^13^C isotope analysis (van der Meer et al., 2001). This bacterium had been shown to synthesize PHB, PHV, and PHB-co-PHV copolymers under photoautotrophic cultivation, where H_2_/CO_2_ (80:20) was fed continuously at 26 mg of carbon supplied/min (van der Meer et al., 2001). This bacterium is thought to fix CO_2_ using the 3-HP pathway (Strauss and Fuchs, 1993).

### 4.2 Cyanobacteria

Cyanobacteria (blue-green algae) are promising photoautotrophic hosts that produce various bioproducts, including organic acids, alcohols, fatty acids, bioplastics precursors, and biofuels (Bühler and Lindberg, 2023). As discussed earlier, cyanobacteria can flourish well with the help of CO_2_ fixation from the atmosphere by the CBB pathway. Some cyanobacterial species can tolerate even high concentrations of CO_2_ (i.e., *Chlorella pyrenoidosa, C. vulgaris, Scenedesmus obliquus, Thermosynechococcus elongatus,* and *Rhodovulum viride*). Their CO_2_ fixation ability mainly depends on the physical parameters, including pH, temperature, light intensity, cultivation mode, and type of bioreactors (Salehizadeh et al., 2020; Ray et al., 2023). During the nitrogen and phosphorous limitation in the growth environment, these photoautotrophs can synthesize a range of intracellular polymers, including glycogen, PHAs, cyanophycin, and polyphosphate (Bühler and Lindberg, 2023). Among these, glycogen and PHAs are carbon-rich energy storage biopolymers; more specifically, glycogen metabolism is conserved in all cyanobacteria. Glycogen biosynthesis is vital in maintaining cellular homeostasis and protecting against environmental stresses. At the same time, PHAs serve as long-term carbon reserves and contribute to managing environmental stress conditions (Bühler and Lindberg, 2023).

**FIGURE 6:**
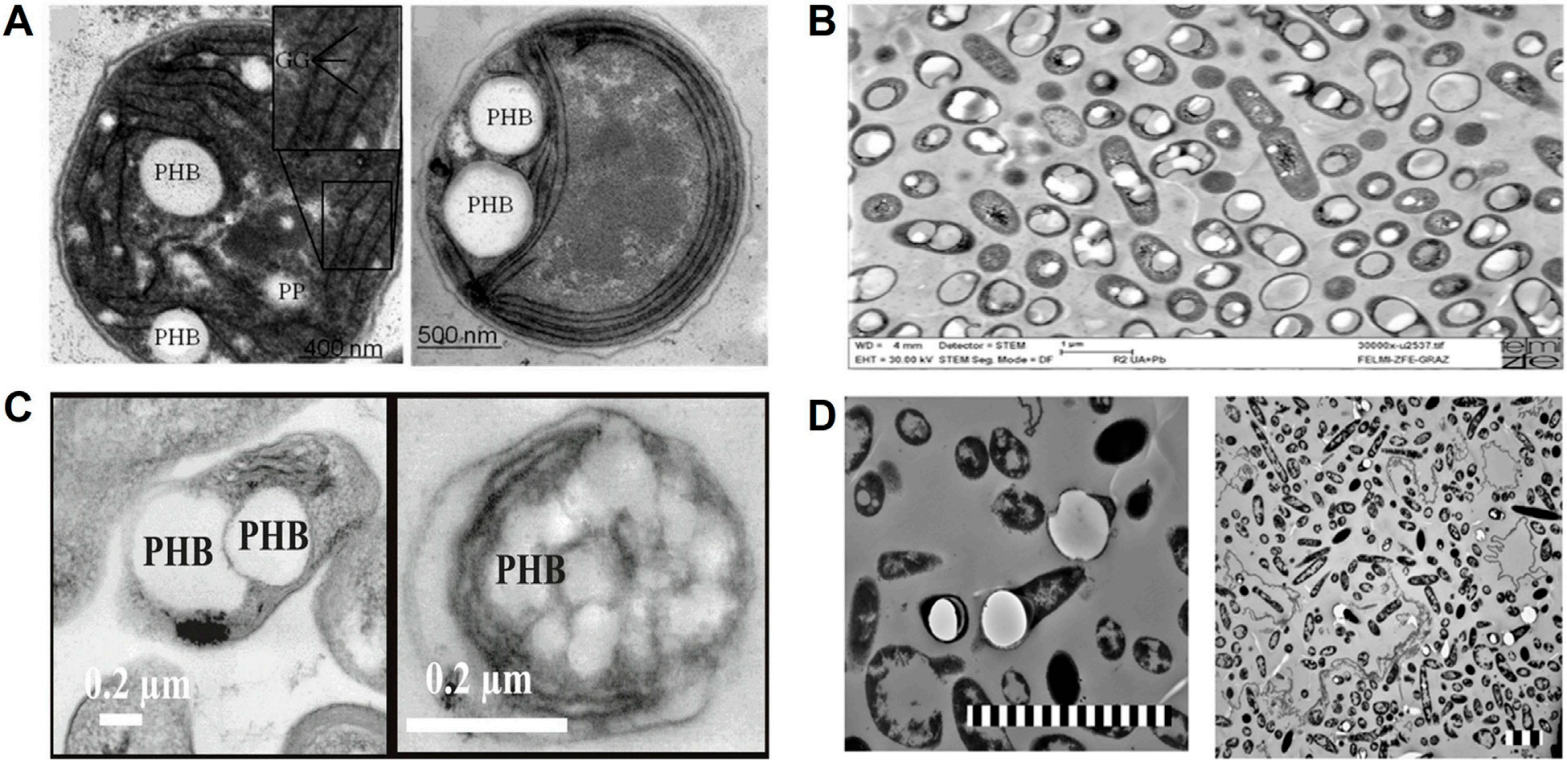
Transmission electron microscopy (TEM) images of autotrophic microorganisms involved in PHB production. **(A)** Photoautotrophic *Synechocystis* sp. PCC 6803 synthesizing PHB using 0.03%–3% v/v CO_2_; GG: glycogen granule, PP: polyphosphate (Damrow et al., 2016). **(B)** Chemolithoautotrophic *C. necator* DSM 545 displaying intracellular PHA granules (bright inclusions), imaged at ×30,000 magnification; scale bar: 1 μm (Koller, 2017). **(C)** PHB synthesis by *R. palustris* TIE-1 under photoautotrophic conditions using N_2_/CO_2_ (80%/20%) and +100 mV vs standard hydrogen electrode (SHE) via microbial electrosynthesis. TEM images (scale bar: 0.2 μm) show cells grown under photoferroautotrophic conditions with Fe(II) and photoelectroautotrophic conditions with a poised electrode, respectively (Ranaivoarisoa et al., 2019). **(D)** Genetically modified acetogen *C. coskatii* [p83_PHB_Scaceti] cultivated on syngas, showing PHB granules; scale bar: 3 μm Copyright permission was obtained from the publisher to reproduce this image (Flüchter et al., 2019).

Cyanobacterial PHA occurrence was first documented in *Chlorogloea fritschii* under mixotrophic conditions (autotrophic and heterotrophic), where NaHCO_3_ and acetate were used together in the growth medium. (Carr, 1966). The predominant PHA producers are *Anabaena, Aphanocapsa, Arthrospira, Calothrix, Chrococcus, Gleocapsa, Lyngbya, Mychrochaete, Nostoc, Phormidium, Synechocystis, Synechococcus, Spirulina,* and *Scytonem*a (Troschl et al., 2017a; Costa et al., 2018; Carpine et al., 2020; Bühler and Lindberg, 2023). All these genera accumulate only PHB while growing on NaHCO_3_ or CO_2_ (Costa et al., 2018). A detailed list of cyanobacteria that produce PHA under autotrophic growth is presented in Table 2. Photoheterotrophic PHB synthesis was also confirmed in *Spirulina* LEB18, wherein sodium acetate and glucose were mainly used as carbon sources. However, photoautotrophic media with NaHCO_3_ showed the highest PHB yield of about 44% compared to photoheterotrophic and mixotrophic conditions (Martins et al., 2014). Under nitrogen and phosphorous-limited conditions, wild-type *Synechocystis* sp. PCC 6714 has produced 16.4% of PHB (in dry cell weight) from CO_2_ (Kamravamanesh et al., 2017). Similarly, continuous aeration and CO_2_ addition have increased the PHB level to 21.5% in *Nostoc muscorum* under a phosphate-starved medium (Haase et al., 2012). Filamentous cyanobacterium *Arthrospira subsalsa* can produce up to 14.7% PHB from CO_2_ under high alkaline conditions (5% NaCl) (Shrivastav et al., 2010). In contrast, thermophilic cyanobacterium *Synechococcus* MA19 was reported to synthesize 55% of PHB while growing in a phosphate-limited autotrophic medium (Nishioka et al., 2001). However, most non-thermophilic cyanobacteria can synthesize only 2%–20% of PHB in the presence of CO_2_ (Troschl et al., 2017a; Costa et al., 2018). Transmission electron microscopic images clearly show that the autotropic synthesis of PHB from the wild-type *Synechocystis* sp. PCC 6803 using CO_2_ (0.03%–3%) as a sole carbon source (Damrow et al., 2016) (Figure 6A). The volumetric productivity of PHB (g/L) under photoautotrophic cultivation was not reported precisely in any of these studies except in *Synechocystis* sp. PCC 6803 with 16–27 mg/L (Monshupanee and Incharoensakdi, 2014) and *Caltorix scytonemicola* TISTR 8095 up to 356.5 mg/L of PHB (Kaewbai-ngam et al., 2016), respectively. So far, the *C. scytonemicola* TISTR 8095 strain has only shown a higher PHB yield under photoautotrophic cultivation. More interestingly, *C. scytonemicola* is thought to produce PHB by nitrogen fixation and CO_2_ reduction. However, this productivity is comparatively less than the commercial autotrophic PHA producer *C. necator*, which can produce up to 61 g/L of PHA using CO_2_ as a carbon source (Salehizadeh et al., 2020; Panich et al., 2021).

**TABLE 2:** Photoautotrophic synthesis of PHA from cyanobacteria.

Cyanobacterial strain	Carbon source	PHA content in DCW (%)	PHA titre (mg/L)	Limiting factor	PHA composition	Bioreactor	References
*Anabaena cylindrica* 10 C	^*a*^	0.2	-	N	PHB	Erlenmeyer Flask	Lama et al. (1996)
*Anabaena* sp.	^*a*^	-	2.3	-	PHB	Erlenmeyer Flask	Kadiyala (2014)
*Arthrospira jenneri* NK1	CO_2_	0.38	3.8	-	PHB	Erlenmeyer Flask	Sili et al. (1990)
*Arthrospira laxissima* MG5	^*a*^	0.3	3.0	-	PHB	Erlenmeyer Flask	Sili et al. (1990)
*Arthrospira maxima*	^*a*^	1.2	-	P	PHB	Erlenmeyer Flask	De Philippis et al. (1992)
*A. maxima*	^*a*^	0.7	-	N	PHB	Erlenmeyer Flask	De Philippis et al. (1992)
*Arthrospira platensis*	CO_2_ (5%)	6	0.8	-	PHB	Erlenmeyer Flask	Campbell et al. (1982)
*A. platensis*	^*a*^	3.5	-	P	PHB	Erlenmeyer Flask	Panda et al. (2005)
*Arthrospira* sp. LEB 18	NaHCO_3_	30.7	150	N, P	PHB	Erlenmeyer Flask	Vanessa et al. (2015)
*Arthrospira subsalsa*	^*a*^	7.45	147	N	PHB	Erlenmeyer Flask	Shrivastav et al. (2010)
*Aulosira fertilissima*	^*a*^	10.5	32.6	P	PHB	Erlenmeyer Flask	Samantaray and Mallick (2012)
*A. fertilissima*	^*a*^	9.8	25.8	N	PHB	Erlenmeyer Flask	Samantaray and Mallick (2012)
*Caltorix scytonemicola* TISTR 8095	^*a*^	25.4	356.5	N	PHB	Erlenmeyer Flask	Kaewbai-ngam et al. (2016)
*Chlorogloeopsis fritschii* PCC 6912	^*a*^	^*b*^	-	N	PHB	Erlenmeyer Flask	Hai et al. (2001)
*Cyanothece* sp. PCC 7424	^*a*^	^*b*^	-	N	PHB	Erlenmeyer Flask	Hai et al. (2001)
*Cyanothece* sp. PCC 8303	^*a*^	0.3	-	N	PHB	Erlenmeyer Flask	Hai et al. (2001)
*Desmonostoc muscorum* SAG 1453-12b *(Nostoc muscorum Agardh)*	CO_2_ (10%)	22.6	248	-	PHB	CSTR	Bhati and Mallick (2016)
*D. muscorum* SAG 1453-12b	^*a*^	21.5	105.4	P	PHB	Erlenmeyer Flask	Haase et al. (2012)
*Gloeocapsa* sp. PCC 7428	^*a*^	<0.3	-	N	PHB	Erlenmeyer Flask	Hai et al. (2001)
*Gloeothece membranacea* PCC 6501	CO_2_ (0.5%)	-	-	-	PHB	Erlenmeyer Flask	Rippka et al. (1971)
*Gloeothece* sp. PCC 6501	^*a*^	^*b*^	-	N	PHB	Erlenmeyer Flask	Hai et al. (2001)
*Gloeothece* sp. PCC 6909	^*a*^	2.5	-	-	PHB	Erlenmeyer Flask	Stal (1992)
*Gloeothece tepidariorum* PCC 6909 *(Synechocystis* sp. CCALA 192*)*	^*a*^	12.5	125	N	PHB	Tubular reactor	Troschl et al. (2018)
*G. tepidariorum* PCC 6909 *(Synechocystis* sp. CCALA 192*)*	^*a*^	6	123	N, P	PHB	Tubular reactor	Meixner et al. (2018)
*Lyngbya* sp. PCC 8106 *(Oscillatoria limosa strain* 23*)*	^*a*^	-	-	-	PHB	Erlenmeyer Flask	Stal et al. (1990)
*Nostoc muscorum*	^*a*^	22.7	30	P	PHB	Erlenmeyer Flask	Panda et al. (2005)
*N. muscorum*	^*a*^	8.58	-	-	PHB	Erlenmeyer Flask	Sharma and Mallick (2005)
*Oscillatoria jasorvensis* TISTR 8980	^*a*^	15.7	-	N	PHB	Erlenmeyer Flask	Kaewbai-ngam et al. (2016)
*Oscillatoria okeni TISTR 8549*	^*a*^	14	103	N	PHB-co-PHV	Erlenmeyer Flask	Taepucharoen et al. (2017)
*Phormidium* sp.	^*a*^	-	7.6	-	PHB	Erlenmeyer Flask	Kadiyala (2014)
*Phormidium* sp. TISTR 8462	^*a*^	14.8	-	N	PHB	Erlenmeyer Flask	Kaewbai-ngam et al. (2016)
*Stanieria* sp. PCC 7437	^*a*^	^*b*^	-	N	PHB	Erlenmeyer Flask	Hai et al. (2001)
*Synechococcus* sp. MA19	CO_2_ (2%)	62	-	P	PHB	Erlenmeyer Flask	Nishioka et al. (2001)
*Synechococcus* sp. MA19	CO_2_ (2%)	55	2,400	P	PHB	Erlenmeyer Flask	Nishioka et al. (2001)
*Synechococcus* sp. MA19	CO_2_ (2%)	27	-	N	PHB	Bottle	Miyake et al. (1996)
*Synechococcus* sp. MA19	^*a*^	0.5	-	N	PHB	Erlenmeyer Flask	Hai et al. (2001)
*Synechocystis* PCC 6803	^*a*^	26	-	N	PHB	Erlenmeyer Flask	Dutt and Srivastava (2018)
*Synechocystis* sp.	^*a*^	5.04	-	N	PHB	Photo-bioreactor	Rueda et al. (2020)
*Synechocystis* sp.	NaHCO_3_	31	241		PHB	Erlenmeyer Flask	Gracioso et al. (2021)
*Synechocystis* sp. PCC 6714	CO_2_ (2%)	20.4	652	N, P	PHB	CSTR-one step process	Kamravamanesh et al. (2019)
*Synechocystis* sp. PCC 6714	CO_2_ (2%)	16.4	342	N, P	PHB	CSTR	Kamravamanesh et al. (2017)
*Synechocystis* sp. PCC 6714	CO_2_ (2%)	14	297	N, P	PHB	CSTR	Kamravamanesh et al. (2018)
*Synechocystis* sp. PCC6803	^*a*^	∼3	-	N	PHB	Erlenmeyer Flask	Wu et al. (2002)
*Synechocystis* sp. PCC6803	^*a*^	11.2	-	P	PHB	Erlenmeyer Flask	Panda and Mallick (2007)
*Synechocystis* sp. PCC6803	^*a*^	9.5	-	N	PHB	Erlenmeyer Flask	Panda and Mallick (2007)
*Synechocystis* sp. PCC6803	^*a*^	4.1	-	N	PHB	Erlenmeyer Flask	Wu et al. (2001)
*Synechocystis* sp. PCC6803	CO_2_ (1%)	3	-	N	PHB	Erlenmeyer Flask	Sudesh et al. (2002)
*Thermosynechococcus elongatus* BP-1	^*a*^	14.5	-	-	PHB	Photo-bioreactor	Eberly and Ely (2012)
*Trichodesmium thiebautii* ^78^	^*a*^	2.3	-	-	PHB	-	Siddiqui et al. (1992)

^a^ Atmospheric air CO_2_ (0.04%).

^b^ Trace amount.

Abbreviations: N, Nitrogen (inorganic nitrogen source); P, phosphorous; NaHCO_3,_ sodium bicarbonate; CSTR, continuous stir tank reactor.

Adding carbon sources like glucose, fructose, acetate, propionate, and valerate into the growth medium can achieve higher PHB content. Such photoheterotrophic condition immensely increased the PHA synthesis in *Nostoc Muscorum* Agardh (Bhati and Mallick, 2015) and *Aulosira fertilissima* (Samantaray and Mallick, 2012) up to 78% (PHB-co-PHV) and 85% (PHB) with volumetric productivity of 0.438 and 1.59 g/L, respectively. Introducing organic substrates causes a metabolic shift from autotrophic to heterotrophic growth. PHA copolymer synthesis under autotrophic conditions is not a feature of cyanobacteria. However, *Anabaena spiroides* TISTR 8075 was found to synthesize the PHB-co-PHV copolymer using CO_2_ as the sole substrate (Tarawat et al., 2020). The same strain has also produced PHB-co-PHV copolymer under mixotrophic cultivation, where acetate, propionate, and valerate were supplemented with CO_2_ (Tarawat et al., 2020). Like this, *Oscillatoria okeni* TISTR 8549 was found to synthesize 9%–14% PHB-co-PHV in their dry cell weight with 4.3–5.5 mol% of HV incorporation under nitrogen-limited photoautotrophic conditions (Taepucharoen et al., 2017). Photo-mixotrophic production of PHA from cyanobacteria is presented in the Supplementary Table S1. Nevertheless, photoheterotrophic growth did not improve the PHB-co-PHV accumulation, which suggests that PHA synthesis in cyanobacteria is strain-specific rather than the type of carbon source used (Taepucharoen et al., 2017).

The feast and famine strategies were also suggested for selecting autotrophic cyanobacterial mixed microbial culture (MMC) for PHB synthesis. The MMC was shown to produce PHB when the sequencing batch reactor (SBR) was entirely void of nitrogen (Arias et al., 2018a). Recent studies have intensified the autotrophic PHA synthesis in pilot-scale closed bioreactor (30L) using MMC mainly composed of cyanobacterial (abundance 60%–70%) species such as *Aphanocapsa* sp. and *Chroococcidiopsis* sp. The cyanobacterial MMC had undergone nitrogen and phosphorus limitation, resulting in 50 and 104 mg/L of PHB on photoautotrophic cultivation’s ninth and eighth day, respectively (Arias et al., 2018b). Most fermentation experiments were conducted in sterile laboratory environments, with very few reports on pilot-scale production of PHB under non-axenic settings. Austrian researchers have developed a 200-L photobioreactor (tubular) and cultured the *Synechocystis* sp. CCALA192 using CO_2_ under non-axenic conditions for over 75 days, with different growth cycles. After 16–20 days, *Synechocystis* sp. CCALA192 produced 1.0 g/L of biomass with 12.5% of PHB (Troschl et al., 2018). Similarly, another Austrian power company (Energie-Versorgung Niederosterreich AG) installed a small pilot-scale photobioreactor (tubular) to produce cyanobacterial biomass. It was subsequently processed for PHB extraction, and the residual biomass was allowed to produce biogas. Their initial results and theoretical calculations suggest that 1 ton of CO_2_ can be converted to 115 kg of PHB and 330 m^3^ of biogas, wherein 700 m^2^ of land may need to make 1 ton of PHB since the land area is one of the crucial factors for economic production (Zhang, 2015). Such numbers are promising for the sustainable production of PHA from cyanobacteria using CO_2_. Therefore, various large-scale cultivation strategies must be developed and assessed for autotrophic PHA production to achieve a high yield.

## 5 Chemolithoautotrophic PHA synthesis from CO_2_

Some procaryotic microorganisms obtain energy by oxidizing or reducing the inorganic compounds (electron donors) such as H_2_, H_2_S, Fe^2+^, CO, NO_3_, and NH_3_, thereby utilizing such energy to fix atmospheric CO_2_ via the CBB pathway. Those microorganisms are collectively called as chemolithotrophs, and most of the chemolithotrophs are obligate autotrophs. Facultative chemolithoautotrophs can adjust their biosynthetic pathways, enabling them to switch between autotrophic and heterotrophic lifestyles. One such example is hydrogen-oxidizing *C. necator*. Autotrophic PHA synthesis has been identified in hydrogen-oxidizing bacteria, acetogens, CO-oxidizing bacteria, sulphur-oxidizing, and nitrite-oxidizing bacteria. This section discusses recent advancements and progress in autotrophic PHA synthesis from CO_2_ by chemolithotrophic wild-type bacteria.

### 5.1 Hydrogen-oxidising bacteria

Hydrogen-oxidizing bacteria can only convert hydrogenous gas mixtures (H_2_, CO, CO_2,_ and CH_4_) to bioproducts. Some facultative or obligate chemolithotrophic bacterial genera, including *Cupriavidus, Comamonas, Ideonella,* and *Pseudomonas*, are known for hydrogen oxidation and autotrophic PHA synthesis. All these bacteria are found to be resistant or tolerant to certain levels of CO; hence, they are also collectively known as CO-oxidizing bacteria. *Cupriavidus* is a well-studied genus for autotrophic PHA synthesis, where CO_2_ is a primary carbon source (Srisawat et al., 2022b; Ray et al., 2023). One of the best species is the *C. necator* H16, is a Gram-negative, non-pathogenic β-proteobacterium and facultative chemolithotroph, which oxidizes the H_2_ and assimilates the CO_2_ via the CBB pathway (Morlino et al., 2023), where O_2_ is an electron acceptor. This bacterium can naturally synthesize the PHAs up to >50% of its dry cell biomass on various carbon sources by autotrophic and heterotrophic routes (Ishizaki et al., 2001; Li and Wilkins, 2020; Behera et al., 2022; Morlino et al., 2023) (Figure 6B). Sometimes, this bacterium can accumulate up to 90% of PHA when the growth medium is amended with anaerobic digestate and 1% acetate (Passanha et al., 2013).

Over time, autotrophic cultivation of *C. necator* using H_2_ has gradually evolved since H_2_ is an insoluble and highly explosive gas substrate (Ishizaki et al., 2001). Two different cultivation systems have been developed to increase PHB production in *C. necator*, such as dead-end and recycled gas culture (Ishizaki et al., 2001). Dead-end cultivation is a process where the gas supply is not continuously replenished, which faces challenges with the gas-to-liquid mass transfer because it needs more aeration (Bongers, 1970). In contrast, the recycled gas closed circuit cultivation method offers several advantages, including continuous gas supply, operational safety, and reduced substrate gas loss (Schlegel et al., 1961; Kodama et al., 1975). Many researchers have explored the theoretical foundations, methodologies, stoichiometry, and realistic bioprocess systems for producing PHB from *C. necator* using CO_2_ as a substrate (Ishizaki and Tanaka, 1990; Tanaka and Ishizaki, 1994; Takeshita and Ishizaki, 1996; Sugimoto et al., 1999). Their studies also explored possible fermentation platforms for this bacterium from a manufacturing standpoint, focusing on challenges like the risk of detonation and inefficient gas utilization caused by exhaust gas flow from the bioreactor. While using an explosion-proof continued stir tank reactor (CSTR), researchers have successfully achieved a high cell density culture of *C. necator* with a yield of 91.3 g/L of dry biomass and 61.9 g/L of PHB (1.55 g/L/h) under autotrophic condition, wherein O_2_ was the limiting factor (Ishizaki et al., 1993; Tanaka et al., 1995). Later, a two-stage cultivation system was introduced along with carboxymethyl cellulose to enhance the mass transfer coefficient within an air-lift fermenter, which produced 56.4 g/L of PHB (0.613 g/L/h) from 69.3 g/L of biomass (Taga et al., 1997). Recently, high-pressure fermentation approaches have also been suggested to increase the gas-to-liquid mass transfer and avoid explosions during gas fermentation. Operating the reactors under elevated pressure from 1.5 to 3 bar, along with the O_2_ limitation, enables a lengthy exponential growth and further boosts the autotrophic PHB production from 10.8 g/L to 29.6 g/L (0.45 g/L/h), respectively (Vlaeminck et al., 2024). A comparison of PHA productivity among the *C. necator* autotrophic studies has been presented in Table 3. Bioengineering aspects of recycled gas systems have also been explored to develop an efficient bioprocess method for industrial cultivation of *C. necator* using inexpensive and instantly accessible gas substrates for the autotrophic production processes. Such studies have shown that high O_2_ levels may inhibit the specific growth rate of *C. necator*, whereas lower gas concentrations could stimulate PHB production (Darani et al., 2006).

**TABLE 3:** Autotrophic PHA production from chemolithoautotrophic bacteria using CO_2_ and other gas mixtures.

Bacterial strain	Gas mixture H_2_:O_2_:CO_2_ (vol%)	Biomass (g/L)	PHA (g/L)	PHA % in DCW	PHA productivity (g/L/h)	Limiting factor	PHA composition	Bioreactor	References
*Beggiatoa* sp. 35Flor	^a^	-	-	-	-	-	-	Shake flask	Schwedt et al. (2012)
*Cupriavidus necator* ACM 1296	70:20:10	16	∼8	∼50	∼0.2	O_2_	PHB	CSTR	Darani et al. (2006)
*C. necator* ATCC 17697	83.0:5.3:10.6^b^	27.3	15.2	55.7	0.684	O_2_	PHB	CSTR	Tanaka and Ishizaki (1994)
*C. necator* ATCC 17697	86.5:4.9:9.8^b^	26.3	21.6	82.1	0.556	O_2_	PHB	CSTR	Tanaka and Ishizaki (1994)
*C. necator* ATCC 17697	84.1:6.7:10.3^b^	42.5	23.9	56.3	0.906	O_2_	PHB	CSTR	Tanaka and Ishizaki (1994)
*C. necator* ATCC 17697	75:15:10	27	16	59	0.225	N	PHB	CSTR	Ishizaki and Tanaka (1991)
*C. necator* ATCC 17697	75:15:10	60	36	60	0.6	O_2_	PHB	CSTR	Ishizaki and Tanaka (1991)
*C. necator* ATCC 17697	85:5:10^c^	58.8	46.2	78.6	0.55	O_2_	PHB	Air-lift	Taga et al. (1997)
*C. necator* ATCC 17697	85:5:10^d^	60	49.2	82	0.41	O_2_	PHB	Air-lift	Taga et al. (1997)
*C. necator* DSM 545	84:2.8:13.2^e^	18	13	72	0.187	N, O_2_	PHB	CSTR	Garcia-Gonzalez et al. (2015)
*C. necator* DSM 545	84:2.8:13.2^e^	46	28	61	0.168	N, O_2_	PHB	CSTR	Garcia-Gonzalez et al. (2015)
*C. necator* ATCC 17697	85:5:10^f^	69.3	56.4	81.4	0.613	O_2_	PHB	Air-lift	Taga et al. (1997)
*C. necator* ATCC 17697	90:6.9:10	85.7	61.5	71.7	1.37	O_2_	PHB	CSTR	Ishizaki et al. (1993)
*C. necator* ATCC 17697	7:1:1: 91(N_2_)	-	0.88	-	-	N	PHB	-	Park et al. (2014)
*C. necator* ATCC 17697	-	5.8	3.65	63	0.076	N	PHB-co-PHV-co-PHHx	Erlenmeyer Flask	Volova et al. (2008)
*C. necator* ATCC 17697	85.2:6.3:8.3	91.3	61.9	67.8	1.55	O_2_	PHB	CSTR	Tanaka et al. (1995)
*C. necator* ATCC 17699	60:20:10	18	14	78	0.233	N	PHB	CSTR	Sonnleitner et al. (1979)
*C. necator* B-10646	70:20:10	48	40.8	85	0.582	N	PHB	CSTR	Volova et al. (2013)
*C. necator* B-5786	60:20:10	18	11.34	63	0.157	N	PHB-co-3HV	CSTR	Volova and Kalacheva (2005)
*C. necator* B-5786	60:20:10	30	22	75	0.314	N	PHB	CSTR	Volova and Voinov (2003)
*C. necator* B-5786	-	6.1	3.74	61.4	0.078	N	PHB-co-PHV-co-PHHx	Erlenmeyer Flask	Volova et al. (2008)
*C. necator* B-5786	60:20:10	12	7.56	63	0.105	N	PHB	CSTR	Volova et al. (2004)
*C. necator* DSM 545	84:2.8:13.2^f^	27	11	41	0.116	N, O_2_	PHB	CSTR	Garcia-Gonzalez et al. (2015)
*C. necator* DSM 545	84:2.8:13.2^f^	21	16	74	0.252	N, O_2_	PHB	CSTR	Garcia-Gonzalez et al. (2015)
*C. necator* DSM 545	84:2.8:13.2^g^	38	13	34	0.109	N	PHB	CSTR	Garcia-Gonzalez and De Wever (2017)
*C. necator* DSM 545	84:2.8:13.2^g^	21	15.3	73	0.225	N	PHB	CSTR	Garcia-Gonzalez and De Wever (2017)
*C. necator* DSM 545	84:2.8:13.2^h^	38	24	63	0.108	N	PHB	CSTR	Garcia-Gonzalez and De Wever (2017)
*C. necator* PAS832	-	-	-	54.4	-	-	PHB, MCL PHAs^k^	Erlenmeyer Flask	Nangle et al. (2020)
*C. necator*	70:20:10	5	3.35	67	0.052	N, O_2_	PHB	PBR	Lu and Yu (2017)
*C. necator* ATCC 17697	86.5:6.5:10^i^	22.9	12.6	55	0.152	O_2_	PHB	CSTR	Sugimoto et al. (1999)
C. necator DSM 545	76.5:3.5:5:15 (N2)	-	29.6	76.1	-	O_2_	PHB	CSTR	Vlaeminck et al. (2024)
*Ideonella* sp*.* strain O-1	70:10:10	6.75	5.26	77.92	0.219	N	PHB	CSTR	Tanaka et al. (2011)
*Nitrobacter winogradskyi*	^a^	-	-	-	-	-	PHB	-	Van Gool et al. (1971)
*Pseudomonas carboxydohydrogena* Z-1062	60:10:10:20 (CO)	17.36	10.08	58	0.18	N, S	PHB	CSTR	Volova et al. (2015)
*P. carboxydohydrogena* Z-1062	60:10:10:20 (CO)	18.48	10.64	57.6	0.19	N	PHB	CSTR	Volova et al. (2015)
*P. carboxydohydrogena* Z-1062	60:10:10:20 (CO)	20.16	12.32	61	0.22	S	PHB	CSTR	Volova et al. (2015)
*Paracoccus denitrificans* NBRC 13301	80:5:10	∼7	∼4	57.3	-	N	PHB	CSTR	Tanaka et al. (2016)

^a^ Atmospheric air CO_2_ (0.04%).

^b^ Heterotrophically grown on fructose, PHB, is produced autotrophically using CO_2_.

^c^ 0.1% sodium carboxymethylcellulose (CMC) amended condition.

^d^ 0.05% CMC, amended condition.

^e^ Heterotrophically grown on glycerol, PHB, is produced autotrophically using CO_2_.

^f^ Heterotrophically grown on glucose, PHB, is produced autotrophically using CO_2_.

^g^ Heterotrophically grown on glucose, PHB, is produced using industrial CO_2_ off-gases from a biogas plant.

^h^ Heterotrophically grown on glucose, PHB, is produced using industrial CO_2_ off-gases from a biorefinery plant.

^i^ Heterotrophically grown on acetate, PHB, is produced autotrophically using CO_2_.

^i^<0.1%–4%.

^j^ PHH, PHO, PHD, PHDD, and PHTD.

Abbreviations: N, Nitrogen (inorganic nitrogen source); P, phosphorous; S, sulphur; CSTR, continuous stir tank reactor; PBR, photobioreactor.

PHB is a well-known SCL-PHA. However, its commercialization has some practical difficulties since this polymer is highly crystalline with high rigidity, brittleness, and low tensile power (Muneer et al., 2020). Such features can be enhanced by integrating different PHA monomers (Sudesh et al., 2000). For example, PHB-co-PHV copolymers have better flexibility and durability than PHB (Reddy et al., 2003; Philip et al., 2007). Moreover, MCL-PHAs and their copolymers are more elastomeric than SCL-PHAs (Anjum et al., 2016). Therefore, synthesizing different PHA copolymers from *C. necator* is inevitable, which can improve the polymer properties and applications. Recent studies have focused on mixotrophic PHA synthesis, where CO_2_ and other PHA precursors were supplied as carbon sources (Supplementary Table S2). For instance, the pulse feeding of valerate to the autotrophic medium effectively incorporated the valerate monomers and produced PHB-co-PHV from *C. necator* (Volova and Kalacheva, 2005; Park et al., 2014). In addition, MCL monomers were also incorporated while adding the MCL precursors (heptanoate, octanoate, and hexanoate) along with CO_2_ (Volova et al., 2008; 2013). Most autotrophic studies with CO_2_ have shown that either nitrogen or O_2_ limitation is a crucial factor for the PHB synthesis in *C. necator* (Panich et al., 2021; Morlino et al., 2023). Internal remobilization of PHB polymers is also observed when the bacterium faces a carbon-deficient condition, which is very common in all polymer-producing microorganisms in nature.

Valorisation of industrial exhaust gas (mainly CO, CO_2,_ and H_2_) is an exciting subject for autotrophic PHA synthesis. However, CO-resistant strains can only tolerate such toxic gas composition since CO is lethal to most bacteria except CO-oxidizers. It has been found that the *C. necator* B5786 strain can exceptionally tolerate 5%–25% (v/v) of CO and produce 70%–75% of PHB-co-PHV copolymer under autotrophic conditions. The PHB-co-PHV polymer also had material properties like those produced from autotrophic fermentation using electrolytic H_2_ (Volova et al., 2002). Most of the wild-type *C. necator* lacks the CO dehydrogenase (CODH). Hence, *C. necator* cannot utilize the CO-containing syngas for PHA synthesis. Researchers have immobilized the CODH enzyme on the *C. necator* cell surface to overcome this, effectively utilizing CO-containing syngas and producing 14.2 g/L of PHB (Shin et al., 2021). Aerobic CO-oxidizing/H_2_-oxidising bacterium *Pseudomonas carboxydohydrogena* Z-1062 (formerly known as *Seliberia carboxydohydrogena* Z-1062) have been studied under autotrophic batch cultivation with a mixture of CO, H_2_, CO_2_, and O_2_ (Volova et al., 2015). This bacterium was shown to synthesize the PHA up to 52.6%–62.8% in dry cell biomass after 56h of process under the limitations of nitrogen and sulphur. PHA production has maximized as 0.13–0.22 g/L/h even though the medium was amended with 10%–30% CO v/v. The produced PHA comprises mainly PHB (99 mol%) with a small portion of PHV (0.24–0.48 mol%). However, 30% v/v of CO concentration affected the growth rate and cell concentration adversely (Volova et al., 2015). The CO-tolerating H_2_-oxidising bacterium *Ideonella* sp. O-1 has also been isolated from soil, which grows autotrophically by assimilating H_2_, O_2_, and CO_2_ as substrates (Tanaka et al., 2011). This bacterium has been shown to sustain up to 30% (v/v) of O_2_ and 70% (v/v) of CO and can produce 5.26 g/L of PHB from the 6.75 g/L of biomass under autotrophic conditions. Such a high tolerance of CO is highly comparable with well-known H_2_-oxidisers like *C. necator* and *A. latus* since they can tolerate up to 5% (v/v) CO (Tanaka et al., 2011). High tolerance of CO is a promising feature, and these strains can be used to produce PHB polymer from industrial exhaust gas, further boosting the circular economy.

### 5.2 Acetogens

Acetogens are metabolically diverse obligate anaerobes and comprise 23 bacterial genera with more than 100 species (Debabov, 2021). All acetogens can fix the C_1_ gases through the WL pathway (Claassens et al., 2016). During gas fermentation, acetogens can assimilate CO_2_ or CO as a substrate, whereas H_2_ or CO supplies reducing equivalents (Bae et al., 2022). Acetogens perform a series of reactions in the WL pathways to reduce the CO_2_ to acetyl-CoA and later synthesize the acetate as a terminal product. The WL pathway is the highest energy-efficient mechanism for CO_2_ reduction and relates to direct energy storage (Claassens et al., 2016; Bae et al., 2022). Their efficient autotrophic flux for synthesizing acetyl-CoA makes them promising candidates for producing value-added chemicals (i.e., organic acids and alcohol) through autotrophic processes since acetyl-CoA is a primary precursor for many biochemicals (Bae et al., 2022; Flaiz and Sousa, 2024). Among the 100 species, only a few are considered critical biocatalysts for producing biochemicals like butanol, 2,3-butanediol, and ethanol. The major acetate producers are *C. aceticum* (Sim and Kamaruddin, 2008)*, Acetobacterium woodie* (Demler and Weuster-Botz, 2011)*,* and *Moorella thermoacetica* (Daniell et al., 2012). *A. woodie* has been shown to produce 44 g/L of acetate, the highest titre achieved so far under H_2_/CO_2_ conditions (Demler and Weuster-Botz, 2011). In addition, *C. ragsdalei*, *C. ljungdahlii,* and *C. autoethanogenum* were used to make fuel-quality ethanol under autotrophic conditions (Bae et al., 2022). Another set of acetogens, including *Butyribacterium methylotrophicum*, *Eubacterium limosum*, and *C. carboxidivorans*, were explored to synthesize 2,3-butanediol (Michael et al., 2011) and butyrate (Bae et al., 2022) under gas fermentation, where C_1_ gases were used as carbon sources. Despite their promising potential, these organisms are not yet viable for industrial applications due to their slow growth rates and low efficiency in autotrophic production. Various cultivation strategies have been employed in gas fermentation to improve the capacity of acetogenic bacteria to transform C_1_ gases into valuable multi-carbon biochemicals. However, the product collection remains restricted to intrinsic chemicals, predominantly acetate and ethanol. The production of energy-dense compounds, including lipids, long-chain alcohols, and PHAs from C_1_ gases, poses significant challenges in acetogens. This difficulty arises from the energetic limitations of autotrophic growth and the lack of essential enzymes required for synthesizing these complex molecules (Bae et al., 2022). To address these challenges, research has been directed towards rechannelling the WL pathway by genetic and metabolic engineering methods, which will be discussed separately in this review. Recently, two-stage co-cultivation has emerged as a method for producing a broader array of biochemicals. This approach combines acetogenic gas fermentation with an acetate conversion process, thereby developing various products that can be synthesized from CO_2_ (Bae et al., 2022). Acetate-consuming microorganisms can flourish well on acetate and produce acetyl-CoA, a metabolic precursor for various biomolecules. Most acetate-converting bacteria are aerobes; growing them with acetogens in the same reactor is impossible. Therefore, two different fermentations must be conducted using two reactors with various parameters. Two-stage co-cultivation consists of an anaerobic reactor in which acetogens convert C_1_ gases into acetate during the first stage. The generated acetate is moved to an aerobic reactor in the subsequent stage for further transformation. Otherwise, the second stage can occur within the same reactor by modifying the operating parameters to facilitate aerobic growth (Bae et al., 2022). This approach produced acetate from *S. ovata,* where CO_2_ was used as a carbon source. It was later utilized by *E. coli* in the second stage, leading to a PHB productivity of 0.5 g/L (Liu Q. et al., 2015). Similarly, *S. ovata* have been used to convert CO_2_ into acetate (stage 1), which was later used as a substrate to produce PHB from *Cupriavidus basilensis* (stage 2). In the optimized media, *S. ovata* generated 10.4 mmol of acetate (L/day) under a CO_2_ environment. When the stage 1 fermented broth was used as a substrate, *C. basilensis* produced 12.54 mg of PHB (L/h), resulting in a net carbon profit of 11.06% from acetate (Cestellos-Blanco et al., 2021). Other metabolic intermediates from acetogens, including formic acid, have been utilized as a substrate for stage 2 bioprocess (Hwang et al., 2020). Initially, *A. woodii* was used to convert CO into formic acid, and then it was used as a substrate to produce PHB by *Methylbacterium extorquens* AM1 (Hwang et al., 2020). A similar two-stage bioprocess has also been demonstrated with *A. woodii* and *C. necator* H16, in which *A. woodii* produced 3 g/L of acetate using CO_2_ as a substrate, which was later used to produce 0.5 g/L PHB by *C. necator* H16 (Al Rowaihi et al., 2018a).

### 5.3 Other chemolithoautotrophs

The genus *Beggiatoa* contains large, thread-like filamentous bacteria in various sulphur-rich environments, including sediments, springs, and activated sludge. *Beggiatoa* obtains energy by oxidizing inorganic sulphur in the presence of oxygen. Recently, *Beggiatoa* sp. 35Flor isolated from marine environments has been shown to synthesize the PHA inclusion bodies while fixing the atmospheric CO_2_ during the movement between the oxygen-sulphide interface. Under anoxic conditions, PHA inclusion bodies were also remobilized during sulphur respiration (Schwedt et al., 2012). *Nitrobacter winogradskyi*, a well-studied nitrite-oxidizing bacterium, can also synthesize PHB, glycogen, and polyphosphate while growing autotrophically with CO_2_ as a carbon source. This bacterium can also depolymerize the PHB when the medium is depleted with nitrite (Van Gool et al., 1971). Denitrifying and sulphur-oxidizing bacterium *Paracoccus denitrificans* NBRC13301 have been studied for autotrophic growth under aerobic conditions when the culture was fed with a mixture of gases (H_2_/O_2_/CO_2_, 8:1:1). *P. denitrificans* exhibited growth at up to 15% oxygen levels, with an optimal growth concentration of 5%, and accumulated 57.3% w/w of PHB under nitrogen limitation (Tanaka et al., 2016). Iron-oxidizing acidophilic bacterium *Acidithiobacillus ferroxidans* can also fix the CO_2_ via the CBB pathway. This organism is mainly used in the biomining of metals. The ultrathin section of *A. ferroxidans* showed PHB-like inclusion bodies under transmission electron microscopy (TEM) (Matlakowska and Sklodowska, 2007). Although it lacks specific genes for PHA production, it stores glycogen as a carbon reserve material. With its complete genome sequence available, exploring genetic modifications to incorporate the PHA synthesis gene could be intriguing. This could potentially exploit this chemolithoautotrophic bacterium for PHA production.

## 6 Microbial electrosynthesis

Bio-electrochemical systems (BES) have previously been suggested for treating waste streams and recovering valuable products from waste materials. BES systems can be used for biological CO_2_ sequestration using electroactive microorganisms as self-sustaining and economic biocatalysts (Pepè Sciarria et al., 2018; Banu et al., 2019). One such BES method is microbial electrosynthesis (MES), where the CO_2_ is reduced into various organic products by electroactive bacteria (i.e., electrolithoautotrophs or chemolithoautotrophs) in the cathodic chamber. Hence, biocathode development is crucial for MES (Logan et al., 2019). Two types of electron transfer mechanisms have been identified in MES systems: direct and indirect electron transfer. Facultative electrolithoautotrophs can perform direct electron transfer where poised potential only acts as an electron source for CO_2_ reduction. In contrast, chemolithoautotrophs execute the indirect electron transfer, wherein metal ions (i.e., Fe^2+^/Fe^3+^, Mn^2+^/Mn^3+^, etc.), H_2_, formate or NH_3_ are used as diffusible electron carriers/mediators or additional electron donors to fix the CO_2_ via CBB pathway or WL pathway (Logan et al., 2019; Bian et al., 2020). In MES, both pathways can produce acetyl-CoA as a central precursor to produce various multi-carbon organic chemicals such as volatile fatty acids (VFAs) (e.g., formate, acetate, butyrate, valerate), ethanol, butanol, lactate, succinate, and 2,3-butanediol or gaseous substances (H_2_ and CH_4_) (Bian et al., 2020). These products can further act as precursors for biofuels, biopolymers (PHAs), polysaccharides, biomass/protein, and long-chain carboxylates with subsequent multi-step bioconversions (Bian et al., 2020). In MES, PHA can be produced in two different ways such as direct conversion of CO_2_ to PHA by photoautotrophic or chemolithoautotrophic bacteria and indirect conversion of CO_2_ to PHA (multi-step process), wherein CO_2_ is reduced into VFAs, followed its transformation to PHA by MMC (Bian et al., 2020; Stöckl et al., 2020). Direct conversion of CO_2_ to PHA through MES has been demonstrated in photoautotrophs, especially PNSB, and they have been shown to conduct a direct electron transfer, where the poised potential is only used as an electron source (photoelectroautotrophy) (Rengasamy et al., 2017). A comprehensive list of microorganisms producing PHAs by microbial electrosynthesis/electrolysis via CO_2_ reduction is presented Table 4. Photoelectroautotrophic PHB synthesis using N_2_/CO_2_ (80%/20%) has been demonstrated in *R. palustris* TIE-1, where the graphite-based cathode was continually supplied at an electric potential of +100 mV vs*.* standard hydrogen electrode (SHE). The MES system was shown to synthesize 4.48 mg/L and 5.49 mg/L of PHB while fed with NH_4_Cl and N_2_ gas as the nitrogen source, respectively (Ranaivoarisoa et al., 2019) (Figure 6C). When the graphite electrode was modified with an immobilized Prussian blue (i.e., Fe^2+^-based redox mediator), *R. palustris* TIE-1 showed a high electron transfer (mostly reversible redox reaction), which resulted in increased cathodic current density (5.6 ± 0.09 μA/cm^2^) and PHB synthesis (18.8 ± 0.5 g/L) compared to unmodified cathodic experiments (Rengasamy et al., 2017). Similarly, *R. vannielii* and *R. udaipurense* were shown to produce PHB from CO_2_ (2.02% and 0.78% cdw_prot_) when carbon felt was used as a working electrode with an electric supply of +100 mV vs. SHE under N_2_-fixation condition (Conners et al., 2024). In general, N_2_-fixing photoelectroautotrophy enhances the PHB synthesis in PSNB; however, PHB productivity is far less than in photoautotrophic or photoheterotrophic conditions (Rengasamy et al., 2017; Conners et al., 2024).

**TABLE 4:** Autotrophic PHA synthesis by microbial electrosynthesis/electrolysis via CO_2_ reduction.

Microorganism	Carbon source/Strategy	Conditions (Electrode/Poised potential)	PHA Yield (mg/L)	PHA % in DCW	PHA Composition	Limiting factor	Reactor type	References
*R. palustris* TIE-1	N_2_:CO_2_ (80%:20%) Direct conversion	Graphite cathode, +100 mV *vs.* SHE	5.49	-	PHB	N^a^	Seal-type single chamber half-cell reactor	Ranaivoarisoa et al. (2019)
*R. palustris* TIE-1	N_2_:CO_2_ (80%:20%) Direct conversion	Graphite cathode, +100 mV vs SHE	4.48	-	PHB	N	Seal-type single chambered half-cell reactor	Ranaivoarisoa et al. (2019)
*R. palustris* TIE-1	N_2_:CO_2_ (80%:20%) Direct conversion	Modified Graphite cathode with Prussian blue (i.e., Fe^2+^-based redox mediator), +100 mV *vs.* SHE	18.8^b^	-	PHB	-	Seal-type single chambered half-cell reactor	Rengasamy et al. (2017)
*Rhodomicrobium vannielii*	N_2_:CO_2_ (80%:20%) Direct conversion	Carbon felt electrode, +100 mV *vs.* SHE	-	2.02	PHB	N^a^	Seal-type single chambered half-cell reactor	Conners et al. (2024)
*Rhodomicrobium udaipurense*	N_2_:CO_2_ (80%:20%) Direct conversion	Carbon felt electrode, +100 mV *vs.* SHE	-	0.78	PHB	N^a^	Seal-type single chambered half-cell reactor	Conners et al. (2024)
*C. necator* H16	Industrial flue gas with CO_2_ (10%) Direct conversion	Platinized titanium expanded metal electrode, −15 mA *vs.* Ag/AgCl	333 ± 44	43 ± 3	PHB	N^a^	Single chambered half-cell reactor	Langsdorf et al. (2024)
*C. necator* H16	N_2_:CO_2_:O_2_ (85%:10%:5%) Direct conversion	Platinized titanium expanded metal electrode, −15 mA *vs.* Ag/AgCl	347 ± 65	59 ± 1	PHB	N^a^	Single chambered half-cell reactor	Langsdorf et al. (2024)
*C. necator* H16	CO_2_ (100%) Direct conversion	Platinized titanium expanded metal electrode, −15 mA *vs.* Ag/AgCl	275 ± 81	51 ± 4	PHB	O_2_	Single chambered half-cell reactor	Langsdorf et al. (2024)
*C. necator* H16	CO_2_ (100%) for formate synthesis; Formate for PHB synthesis, Two-step process	Indium nanoparticle electrode, −1.2 V *vs.* RHE	25.2	-	PHB	N	Integrated one-pot half-cell system	Al Rowaihi et al. (2018b)
*C. necator* DSM-428	CO_2_ (100%) for formate synthesis; Formate for PHB synthesis, Two-step process	Gas diffusion electrode (90% Sn powder, 5% PTFE and 5% activated carbon), −50 mA cm−2	56	34	PHB	N	Three chambered electrolysis reactor	Stöckl et al. (2020)
*C. necator* DSM-428	CO_2_ (100%) for formate synthesis; Formate for PHB synthesis, Two-step process	Gas diffusion electrode (90% Sn powder, 5% PTFE and 5% activated carbon), 150 mA cm^−2^	63 ± 16	29.1 ± 7.1	PHB	N	Three chambered electrolysis reactor	Dinges et al. (2024)
*Clostridium*-rich mixed microbial culture	CO_2_ (100%) for VFA (butyrate) synthesis; Butyrate for PHB synthesis, Two-step process	Carbon cloth electrode, −0.8 V *vs.* SHE	74.4^c^	-	PHB	N	Two-chambered tubular reactor	Pepè Sciarria et al. (2018)
*R. eutropha* (Rubisco enzymes genetically engineered)	CO_2_ (100%) Direct conversion	Carbon cloth cathode, −0.6 V *vs.* Ag/AgCl	485 ± 13	-	PHB	N	Two-chambered reactor	Chen et al. (2018)
*R. eutropha* (Wild type)	CO_2_ (100%) Direct conversion	Carbon cloth cathode, −0.6 V *vs.* Ag/AgCl	165 ± 8	-	PHB	N	Two-chambered reactor	Chen et al. (2018)
*Kyrpidia spormannii* EA-1	N_2_:CO_2_:O_2_ (77.5:20:2.5%) Direct conversion	Graphite electrode −625 mV *vs.* SHE	26.8^d^	-	PHB	N	6-electrode battery glass reactor, H-cell	Pillot et al. (2022)
*C. necator* DSM-428	CO_2_ (10%) + 4% glycerol	Carbon cloth electrodes, −955 mV for cathode and + 545 mV *vs.* Ag/AgCl at the anode	0.0122 ± .2E^−03^	-	PHB	N	Two-chambered reactor	Nastro et al. (2025)

^b^ Yield as g/L.

^c^ Yield as g PHA ·100 g-1 Volatile Suspended Solid (VSS).

^d^ Yield as μg·cm−2 or 96 mg·day−1 m−2.

^a^ Nitrogen gas.

Abbreviations: N, Nitrogen (inorganic nitrogen source); SHE, standard hydrogen electrode; RHE, reversible hydrogen electrode; PTFE, Polytetrafluoroethylene (Teflon).

Direct conversion of CO_2_ to PHA is also recognized in *C. necator* H16 using industrial flue gas as a CO_2_ source derived from a coal-fired co-generation plant and H_2_ derived from water electrolysis (Langsdorf et al., 2024). Industrial flue gas does not affect the growth and PHB synthesis in *C. necator* H16; following electrochemical CO_2_ reduction has resulted in 333 ± 44 mg/L of PHB (43% ± 3% in dry cell weight) (Langsdorf et al., 2024). Furthermore, a MES-based one-pot carbon capture setup was recently developed to convert CO_2_ to formate (22 mM, at a pH of 7.5), and later the same formate was used to produce PHB by *C. necator* H16. PHB synthesis up to 25.2 mg/L (1.3 mol/h formate uptake) was recognized only when electrolysis functioned in the one-pot carbon capture system within 8h of the electrochemical process under a constant electric supply of −1.2V vs*.* reversible hydrogen electrode (RHE). Reactive oxygen species stress and nitrogen limitation might have triggered the PHB synthesis (as stress mitigation) instead of the co-generation of H_2_ during the electrolysis (Al Rowaihi et al., 2018b). Dinges et al. (2024) have demonstrated a two-step CO_2_ reduction process using a drop-in electrolysis process. Initially, CO_2_ was reduced to formate (441 ± 9 mmol/L) by *C. necator* H16, which further transformed into PHB by the same strain in a fed-batch reactor system, resulting in 63 ± 16 mg/L/OD of PHB (29.1% ± 7.1% in dry cell weight) (Dinges et al., 2024). Despite that, MMC were also used in MES systems for PHA synthesis. Pepè Sciarria et al. (2018) have used *Clostridium*-rich MMC to synthesize VFAs, especially acetate (43 mM carbon/L) and butyrate (103 mM carbon/L). The VFAs were extracted and concentrated, resulting 400 mM carbon/L (∼65% butyrate) was used as a feed to synthesize the PHA (74.4g/100g of volatile suspended solids) by MMC derived from activated sludge and the overall carbon conversion was estimated as 0.14 kg of PHA from 1 kg of CO_2_ (Pepè Sciarria et al., 2018). Though the number of studies on direct or indirect conversion of CO_2_ to PHA by MES is scarce, there are plenty of MES studies for only producing VFAs from CO_2_ using pure and mixed culture (Kumar P. et al., 2018). Researchers also have proposed that producing VFAs from CO_2_ is an indirect way of CO_2_ fixation, and it can be exploited further for PHA production. This approach offers a promising alternative to direct CO_2_ conversion, with potential advantages regarding CO_2_ fixation efficiency, H_2_ utilization, raw material costs, process performance, and safety (Garcia-Gonzalez and De Wever, 2018). Even though many technical factors influence MES productivity, including reactor configurations, cathode materials, microbial stability, and electron transfer mechanisms (Logan et al., 2019; Bian et al., 2020). In addition, MES also has its bottlenecks, mainly high energy consumption and poor CO_2_ assimilation efficiency. Despite all these drawbacks, MES is the best CCU strategy and sustainable route of CO_2_ reduction for producing diverse multi-carbon products (Pepè Sciarria et al., 2018; Banu et al., 2019).

## 7 Genetic engineering approaches for autotrophic PHA synthesis

Many possibilities exist to enhance the autotrophic PHA synthesis in photoautotrophs and chemolithoautotrophs. Tuning CO_2_ fixation pathways, introducing new enzymatic pathways, and overexpressing PHA synthesis enzymes through genetic engineering approaches could enhance CO_2_ bioconversion to PHAs in microbial systems. This chapter comprehensively reviews recent advancements and innovations in the genetic engineering of autotrophic microorganisms to enhance PHA production. A detailed list of genetic modifications and autotrophic PHA synthesis is summarized in Table 5.

**TABLE 5:** Autotrophic PHA production from genetically modified microorganisms.

Microorganism	Genetic modification	Carbon source	Biomass (g/L)	PHA (g/L)	PHA % in DCW	Limiting factor	PHA composition	Bioreactor	References
Photosynthetic bacteria									
*R. rubrum* S1	Overexpression of *PntAB* from *E. coli* MG1655 and phaB1 from *C. necator* H16	Syngas (10% CO_2_, 40% CO, 40% H_2_, 10% N_2_)	-	-	10.1	N	PHB-co-PHV	Erlenmeyer Flask	Heinrich et al. (2015)
*R. rubrum*	Overexpression of ACP, *phaG*, PP_0763, and *phaC1* from *Pseudomonas putida* KT2440	Syngas (10% CO_2_, 40% CO, 40% H_2_, 10% N_2_)	1.0	-	6.7	N	PHD-co-PHO	Erlenmeyer Flask	Daniel et al. (2016)
Cyanobacteria									
*Synechocystis* sp. PCC 6803	*phaE* and *phaC* deleted at S3 site, *Ptac-tesB-kanR* integrated at S2 site, *CmR-Ptac-phaA-phaB* integrated at S4 site	CO_2_ (0.04%)	-	0.533	-	P	3-HB^a^	Erlenmeyer Flask	Wang et al. (2013)
*Synechocystis* sp. PCC 6803	Overexpression *phaEC* from *Microcystis aeruginosa* NIES-843	CO_2_ (2%)	0.206	0.0143	7	-	PHB	Erlenmeyer Flask	Hondo et al. (2015)
*Synechocystis* sp. PCC 6803	Overexpression of *phaABEC* from *C. necator*	CO_2_	-	-	26	N	PHB	Erlenmeyer Flask	Khetkorn et al. (2016)
*Synechocystis* sp. PCC 6803	Overexpression of *phaABEC* from *C. necator*	CO_2_ + 0.4% acetate	-	-	35	N	PHB	Erlenmeyer Flask	Khetkorn et al. (2016)
*Synechocystis* sp. PCC 6803	Overexpression of *sigE* (transcriptional regulator)	CO_2_ (1%)	-	0.014	1.4	N	PHB	Erlenmeyer Flask	Osanai et al. (2013)
*Synechocystis* sp. PCC 6803	Overexpression of *rre37* (response regulator) and *sigE*	CO_2_	-	0.0172	-	N	PHB	Erlenmeyer Flask	Osanai et al. (2014)
*Synechocystis* sp. PCC 6803	Deletion of *agp* (ADP-glucose pyrophosphorylase)	CO_2_	-	-	14.9	N	PHB	Erlenmeyer Flask	Wu et al. (2002)
*Synechocystis* sp. PCC 6803	Deletion of *agp* (ADP-glucose pyrophosphorylase)	CO_2_ + 15 mM acetate	-	-	18.6	N	PHB	Erlenmeyer Flask	Wu et al. (2002)
*Synechocystis sp*. PCC 6803	Overexpression of *xfpk* (phosphoketolase) from *Bifidobacterium breve* and deletion of *pta* (phosphotransacetylase) and *ach* (acetyl-CoA hydrolase)	CO_2_ (2%)	2.42	0.232	12.4	N, P	PHB	PBR	Carpine et al. (2017)
*Synechocystis* sp. PCC 6803	Deletion of *phaEC* and introduction of *tesB* (thioesterase), optimisation of RBS site	CO_2_ (5%)	-	1.84	-	N	PHB	Erlenmeyer Flask	Carpine et al. (2017)
*Synechocystis* sp. PCC 6803	Deletion of *pirC* (regulatory protein), overexpression of *phaA* and *phaB* from *C. necator* (*Δ*pirC-RE*pha*AB)	NaHCO_3_	-	-	63	N, P	PHB	Erlenmeyer Flask	Koch et al. (2020)
*Synechocystis* sp. PCC 6803	Deletion of glycogen synthesis gene glgC (*Δglg*C)	CO_2_ (0.03%–3%)	-	-	13	N	PHB	Erlenmeyer Flask	Damrow et al. (2016)
*Synechocystis* sp. PCC 6714	Random UV mutagenesis (Mt_a24)	CO_2_ (2%)	-	1.16	30	N, P	PHB	CSTR	Kamravamanesh et al. (2019)
*Synechocystis* sp. PCC 6714	Random UV mutagenesis (Mt_a24)	CO_2_ (2%)	∼3	0.735	∼25	N, P	PHB	CSTR	Kamravamanesh et al. (2018)
*Synechocystis* sp. PCC 6714	Knock out of exopolysaccharide (*exo*D) gene	CO_2_ (5%)	-	-	∼16.5	N, P	PHB	Erlenmeyer Flask	Mittermair et al. (2021)
*Synechococcus* sp. PCC 7002	Heterologous expression of *phaABEC* from *Chlorogloeopsis fritschii*, *gbd1* (hydroxybutyrate dehydrogenase), and *cat2* (hydroxybutyryl-CoA transferase) from *Porphyromonas gingivalis*	CO_2_ (1%)	-	-	4.5	N	PHB-co-P4HB	Erlenmeyer Flask	Zhang et al. (2015)
*Synechococcus* sp. PCC 7002	Heterologous expression of *phaCAB* genes from *C. necator* and complementation of *recA* null mutation by *E. coli recA*	CO_2_ (1%)	-	-	52	-	PHB	Erlenmeyer Flask	Akiyama et al. (2011)
*Synechococus elongatus* PCC 7942	Heterologous expressions of *phaAB* from *C. necator, tesB* from *E. coli* and *P. putida*, and *nphT7* (acetoacetyl-CoA synthase) from *Streptomyces* sp	50 mM NaHCO_3_	-	1.22	-	N	PHB	Erlenmeyer Flask	Ku and Lan (2018)
*S. elongatus* UTEX 2973	Heterologous expression of *phaABC* operon from *C. necator*	Industrial flue gas: 3%–6% CO_2_, 11.99% O_2_, 21.72 ppm NOx, 1.43 ppm CO	-	0.42	16.7	N	PHB	PBR	Roh et al. (2021)
*S. elongatus* UTEX 2973	Heterologous expression of *phaABC* operon from *C. necator*	CO_2_ (5%)	**-**	0.278	21	N	PHB	PBR	Roh et al. (2021)
*Nostoc* sp. PCC7120	Heterologous expression of *phaCAB* genes from *C. necator*	5 mM NaHCO_3_	-	-	30	N	PHB	Erlenmeyer Flask	Fink et al. (2025)
Hydrogen-oxidizing bacteria									
*C. necator* H16	Heterologous expression of RuBisCO from *Synechococcus* sp. PCC 7002	H_2_:CO_2_:O_2_ (7:1:1)	-	0.34^b^	34	N	PHB	CSTR	Li et al. (2020)
*C. necator* H16	Heterologous expression of *cox*MSLDEFG from *Oligotropha carboxidovorans* OM5	70% air +30% Syngas (CO:CO:H_2_:N2_2_, 10%:40%:40%:10%)	2.62	1.302	49.7	N	PHB	Erlenmeyer Flask	Heinrich et al. (2015)
*C. necator* H16	Heterologous expression of *pha*C1P from *Pseudomonas* sp. 61–3 s, *pha*ABRe from *C. necator*, ketothiolase (*bkt*B) from *C. necator*	H_2_:O_2_:CO_2_: N_2_ (3.6:7.6:12.3:76.5)		0.14	57	N	PHB-PHV-PH4MV	CSTR	Miyahara et al. (2020)
*C. necator* H16	Heterologous expression of haemoglobin gene (*vgb*) from *Vitreoscilla,* knocking out L-lactate dehydrogenase (*ldh*)	CO_2_:H_2_:O_2_ (1:7:0.25)	0.55	0.277	50.4	O_2_	PHB	Serum Bottles	Tang et al. (2020)
*C. necator* H16 (MF01)	Heterologous expression of (mcl)-specific β-ketothiolase (*bktB)*	H_2_:CO_2_:O_2_ (8:1:1)	8.52	∼7.31	85.8	N	PHB-co-PHHx	Erlenmeyer Flask	Tanaka et al. (2021)
*C. necator *H16 (PAS831)	Overexpression of different thioesterases (TEs) with PHA synthases (phaCs)	H_2_:CO_2_ (8:2)	0.542	0.235	43.4	N	PHAs with C4-C14 monomers	Erlenmeyer Flask	Nangle et al. (2020)
Acetogens									
*Clostridium autoethanogenum*	Heterologous expression of PHA enzymatic pathway (phaCAB genes) from *C. necator*	Syngas with 20% H_2_ (50% CO, 20% CO_2_, 20% H_2_, 10% Argon	-	0.027	5.61	H_2_	PHB	CSTR	(de Souza Pinto Lemgruber et al., 2019)
*C. coskatii*	Heterologous expression of thiolase (*thlA*), CoA-transferase (*ctfA/B*) from *Clostridium acetobutylicum* and (R)-3-hydroxybutyrate dehydrogenase (*bdhA*) from *Clostridioides difficile*	Syngas (10% CO_2_, 40% CO, 40% H_2_, 10% N_2_)	-	0.176	-	-	3HB^a^	Bottle	Flüchter et al. (2019)
*C. coskatii*	Heterologous expression of *thl*A, *hbd* and *crt* from *Clostridium scatologenes*; phaJ and *pha*EC from *Clostridium acetireducens*	Syngas (10% CO_2_, 40% CO, 40% H_2_, 10% N_2_)	-	-	1.12	-	PHB	Bottle	Flüchter et al. (2019)
*A. woodie*	Heterologous expressions of *thl*A, *hbd* and *crt* from *C. scatologenes*; phaJ and *pha*EC from *C. acetireducens*	CO_2_:H_2_ (33% + 67%)	1.23	0.0235	1.9	-	PHB	Bottle	Höfele and Dürre (2023)
*C. ljungdahlii*	Heterologous expression and adaptation of acetate reincorporating pathway	Syngas (10% CO_2_, 40% CO, 40% H_2_, 10% N_2_)	-	3	-	-	3HB^a^	Bottle	Jia et al. (2021)

^a^ 3-hydroxybutyrate (3HB) is the/precursor of PHB.

^b^ PHA, production in g/g.

Abbreviations: N, Nitrogen (inorganic nitrogen source); P, phosphorous; CSTR, continuous stir tank reactor; PBR, photobioreactor.

### 7.1 Tuning of CO_2_ fixation pathways

The CBB pathway is a central metabolism for carbon fixation in photoautotrophs and some chemolithoautotrophs except acetogens. CCB pathway employs many key enzymes to reduce the CO_2_ in cellular biomaterials, including RuBisCO, PrkA, and SBPase. However, RuBisCO could not differentiate O_2_ from CO_2_, and it catalyzes the undesired oxygenation instead of carboxylation during the photorespiration/dark fermentation. To overcome this, cyanobacteria and PNSB execute the CCMs (see section 3), which decrease the overall CO_2_ fixation ability and energy transport of the CBB pathway. Therefore, genetic engineering of the RuBisCO enzyme may open avenues for increased CO_2_ fixation and following high titer of cellular products. Significant efforts have been undertaken to improve RuBisCO’s carboxylation efficiency. For example, creating a point mutation in the larger subunit of the RuBisCO (RbcLF140I) enzyme in *Synechocystis* sp. PCC 6803 has intensified carboxylation activity by 2.9-fold and enhanced the photorespiration rate by approximately 55% (Durão et al., 2015).

In addition, overexpression of RuBP regeneration enzymes also increased the CBB pathway carbon flux, further enhancing the autotrophic ethanol production from *Synechocystis* sp. PCC 6803 (Roussou et al., 2021). The engineering of CCMs has also improved the activity of RuBisCO, leading to significant changes in the HCO_3_^−^ transport systems or the introduction of additional HCO_3_^−^ transport. This enhancement has improved CO_2_ fixation and subsequent biomass production in *Synechocystis* sp. PCC 6803. As a result, there has been a high production of intra (∼50%) and extracellular polymers (3-fold), such as glycogen and exopolysaccharides, respectively (Kamennaya et al., 2015; Gupta et al., 2020). The reductive glycine mechanism was recently proposed to replace the CBB pathway for formate production, thereby enhancing C_1_ assimilation in *C. necator* H16. However, very little growth has been achieved compared to the native CBB pathway. Li et al. (2020) also focused on enhancing the CBB pathway and H_2_ utilization in *C. necator* to increase biomass and PHB synthesis. They achieved this by incorporating the RuBisCO enzyme from *Synechococcus* sp. PCC 7002 into *C. necator* system and adjusting the membrane-bound and soluble hydrogenase expression levels. As a result, the engineered strain showed up to 34% of PHB, in contrast to the wild-type strain (Li et al., 2020). Moreover, heterologous expression of CCMs-related enzymes has been accomplished in heterotrophs like *E. coli* and *Corynebacterium glutamicum* (Panich et al., 2021). Such alteration can be implemented in *C. necator* since this bacterium lacks the CCMs to overcome the RuBisCO inefficiency during the carboxylation phase of the CBB pathway.

### 7.2 Introducing new PHA enzymatic pathways by heterologous expression

PHA-producing model strain *C. necator* H16 does not have CODH by nature. Hence, wild-type strains cannot utilize the CO-containing syngas for PHA production. To overcome this hurdle, *C. nectar* H16 was genetically engineered by heterologous expression of CODH genes from the chemolithoautotrophic bacterium *Oligotropha carboxidovorans* OM5. The modification enabled *C. necator* H16 to use CO and CO_2_, leading to a 1.8-fold increase in biomass over the wild-type strain. PHB synthesis has significantly improved in *C. necator* H16 at 49.7% compared to the wild-type strain, which can synthesize only 40.8% (Heinrich et al., 2015). Most acetogens cannot naturally synthesize PHA; hence, PHA-synthesizing enzymes are required for heterologous production. The PHA synthetic pathway from *C. necator* H16 was expressed into *Clostridium autoethanogenum*. The genetically engineered *C. autoethanogenum* produced 5.58% of PHB by autotrophic gas fermentation while growing on a synthetic gas mixture (syngas) (de Souza Pinto Lemgruber et al., 2019). Flüchter et al. (2019) genetically engineered *C. ljungdahlii* and *C. coskatii* to utilize syngas for autotrophic PHB production. They introduce a novel PHA pathway containing thiolase (*thlA*), (R)-3-hydroxybutyrate dehydrogenase (*bdhA*), and CoA-transferase (*ctfA/B*) into *C. ljungdahlii* and *C. coskatii*. Consequently, *C. coskatii* produced 0.102 and 2.26 g/L of PHB under autotrophic and heterotrophic conditions, respectively. Meanwhile, *C. ljungdahlii* failed to produce PHB even after having new PHA synthetic genes (Flüchter et al., 2019). Similarly, *C. coskatii* was completely engineered with new PHA synthetic pathways, wherein crotonase (*crt*), 3-hydroxy butyryl-CoA dehydrogenase (*hbd*) and thiolase (*thlA*) genes were derived from *C. scatologenes* and PHA synthase (*phaEC*) and (R)-enoyl-CoA hydratase (*phaJ*) genes were derived from *C. acetireducens*, respectively. Subsequently, the engineered *C. coskatii* produced 1.12% PHB under autotrophic conditions using syngas as a substrate (Flüchter et al., 2019) (Figure 6D). The same PHB synthetic pathway was introduced into the acetogen *A. woodie*, which produced 1.9% of PHB during autotrophic cultivation (Höfele and Dürre, 2023). In addition, recombinant *C. ljungdahlii* was shown to synthesize 3-hydroxybutyrate (3-HB) as an unexpected product while incorporating the isopropanol synthetic pathway. Under autotrophic conditions, *C. ljungdahlii* produced 3 g/L of 3-HB along with ethanol (28.4 g/L) and isopropanol (13.4 g/L) (Jia et al., 2021). *S. elongatus* PCC 7942 is an outstanding cyanobacterial strain used for various biochemical production; however, it does not naturally possess a PHA synthesis pathway. However, it can be genetically engineered to synthesize PHA by establishing the mandatory gene cluster from another organism. The PHA synthetic gene cluster from *C. necator* H16 has been introduced into *S. elongatus* PCC 7942, resulting in 25% of PHB under autotrophic conditions (Takahashi et al., 1998). Similarly, *Synechococcus* sp. PCC 7002, also engineered with *a phaABEC* gene cluster obtained from *C. necator* H16, results in autotrophic PHB synthesis of up to 52% (Akiyama et al., 2011). Heterologous expression of the *phaABEC* gene cluster from *Chlorogloeopsis fritschii* PCC 9212 and deletion of the *ccmR* gene in *Synechococcus* sp. PCC 7002 enables the autotrophic synthesis of P (3HB-co-4HB) at 4.5% dry cell weight, with 4HB making up 12 mol% of the copolymer (Zhang et al., 2015). Roh et al. (2021) genetically expressed the whole *C. necator* PHA pathway into the *S. elongatus* UTEX 2973, resulting in PHB synthesis up to 420 mg/L (16.7% w/w) with a yield titer of 46.7 mg/L/d under photoautotrophic cultivation with industrial flue gas as a carbon substrate (Roh et al., 2021).

### 7.3 Overexpression of PHA synthase and other enzymes

Genetic modification, especially overexpression of PHA synthesis enzymes, has been employed in different microorganisms to enhance autotrophic PHA synthesis. Most of the PHA producers synthesize only PHB in autotrophic conditions. Genetically engineered strains can synthesize PHA copolymers. Recently, *C. necator* H16 was engineered with a different set of enzymes, such as monomer supplying gene (*phaABRe*) and 3-keto thiolase (*bktB*) from other C. *necator* strains and PHA synthase 1 (*phaC1P*s) from *Pseudomona*s sp. 61-3. The genetically engineered *C. necator* H16 produced a PHA copolymer of about 0.14 ± 0.05 g/L with 57% of PHB along with 1.2 mol% 3-hydroxyvalerate and 3-hydroxy-4-methyl butyrate (Miyahara et al., 2020). *Pseudomonas* species are known for MCL-PHA synthesis by nature. Hence, MCL-PHA synthetic genes can be overexpressed in autotrophic hosts, including *C. necator*. Tanaka et al. (2021) engineered the *C. necator* with β-ketothiolase gene (*bktB*) encoding MCL-PHAs (C5-C14). They produced PHA copolymer up to 85.8% ± 13.2% from 8.52 ± 1.92 g/L of biomass under autotrophic conditions. The produced PHA copolymers comprise PHB and PHHx monomeric units with 96.7 ± 14 and 3.3 ± 1.4 mol%, respectively (Tanaka et al., 2021). Also, researchers have utilized metabolic engineering strategies to improve the productivity of PHA from CO_2_. For example, *C. necator* was engineered to produce PHA and grow effectively concurrently under autotrophic conditions. Tang et al. (2020) introduced a haemoglobin gene (*vgb*) from *Vitreoscilla* to enhance oxygen usage and knock out L-lactate dehydrogenase (*ldh*) genes in the CBB pathway to channel the carbon flow toward PHA synthesis in *C. necator*, which resulted in 0.55 g/L of biomass with 50.4% PHA under autotrophic condition (Tang et al., 2020).

Several genetic engineering approaches have been applied to photoautotrophs to increase PHA accumulation using CO_2_. A recombinant *R. rubrum* S1 strain, engineered with the *pntAB* gene from *E. coli* MG1655 and the *phaB1* gene from *C. necator* H16, was able to accumulate a PHB-co-PHV copolymer at 5.1% concentration, with a PHV content of 28 mol%, during autotrophic fermentation using syngas (Heinrich et al., 2015). MCL-PHA homopolymers such as 3-hydroxy decanoate (3HD) and 3-hydroxy octanoate (3HO) have also been produced from *R. rubrum* while modifying the wild-type strain with MCL-PHA genes from *Pseudomonas putida*. Such modification resulted in the bioconversion of synthetic syngas to MCL-PHA up to 7% in dry cell weight (Daniel et al., 2016). *Synechocystis* is a natural PHA producer; however, its productivity is far lower than that of other autotrophic hosts. Efforts to overexpress *C. necator* PHA synthase genes (*phaABEC*) in *Synechocystis* sp. PCC 6803 resulted in a strain that produced 26% PHB under nitrogen-limiting conditions (Khetkorn et al., 2016). The same recombinant strain showed 35% PHB production under the mixotrophic conditions with 0.4% acetate as a co-substrate (Khetkorn et al., 2016). Overexpression of the *phaEC* genes from *Microcystis aeruginosa* NIES-843 in *Synechocystis* sp. PCC 6803 significantly boosted PHB accumulation to 7% of dry cell weight, with a productivity of 10.59 mg/L, a 12-fold increase over the wild-type strain (Hondo et al., 2015). Orthwein et al. (2021) developed a novel approach to enhance PHB synthesis using the *Synechocystis* sp. PCC 6803 mutant strain known as PPT1 (Δ*pir*C-RE*phaAB*). Research revealed that the *PirC* protein influences glycolytic carbon flow in a P_II_-reliant behavior, thereby controlling the carbon flux in cyanobacteria (Orthwein et al., 2021). The overexpression of the *phaCAB* gene cluster from *C. necator* in *Synechocystis* sp. PCC 6803 (Δ*pirC*) led to an accumulation of PHB reaching up to 61%, representing a 6.1-fold increase compared to the wild-type strain (Koch et al., 2020). Acetyl-CoA is crucial for central carbon metabolism and PHA synthesis, so higher intracellular levels of acetyl-CoA may enhance PHA production in cyanobacteria. Carpine et al. (2017) demonstrated this concept by engineering a strain of *Synechocystis* sp. PCC 6803. They deleted the *pta* (phosphotransacetylase) and *ach* (acetyl-CoA hydrolase) genes while introducing the *xfpk* (phosphoketolase) gene from *Bifidobacterium breve*. The engineered strain generated 232 mg/L of PHB, 12% of its total weight, yielding 7.4 mg/L/day. The wild-type strain generated only 1.8% PHB, yielding 3.05 mg/L/day (Carpine et al., 2017).

Most cyanobacteria concurrently produce intracellular (i.e., glycogen and PHB) and extracellular polymers (exopolysaccharides). The knockout of one competitive pathway may increase the other polymer production. For example, while disrupting the glycogen production in *Synechocystis* sp. PCC 6803, by creating a glycogen defective mutant strain (Δ*glgC*), surprisingly increased the PHB synthesis up to 13%, whereas the wild-type strain showed only 8% of PHB under autotrophic cultivation (Damrow et al., 2016). Similarly, redirecting glycogen metabolism towards PHB synthesis was accomplished by overexpressing the RNA polymerase sigma factor (*sigE*) in *Synechocystis* sp. PCC 6803. This modification led to a 2.3-fold increase in autotrophic PHB synthesis, reaching approximately 14 mg/L (Osanai et al., 2013). Classical UV random mutagenesis can also be used as an alternative to genetic engineering approaches to generate more efficient PHA producers. Random mutagenesis strangely intensified the autotrophic growth of *Synechocystis* sp. PCC 6714 and increased PHB synthesis up to 37% with a yield of 134.2 mg/L/d/, thereby showing the potential of UV mutagenesis to improve cyanobacteria for efficient CO_2_ uptake and PHA synthesis. In addition, knockout of exopolysaccharide synthesis (Δ*exoD)* in *Synechocystis* sp. PCC 6714, resulting in higher PHB synthesis (∼16.5%) than the control strain (13%) under nitrogen/phosphorous limitation (Mittermair et al., 2021).

## 8 Effect of nutrients on autotrophic PHA synthesis

Autotrophic PHA production using CO_2_ is greatly influenced by the availability of macro and micronutrients essential for microbial metabolism and enzyme activity (Saravanan et al., 2022). Macro-nutrients such as nitrogen, phosphorus, and sulphur are essential for the synthesis of cellular components and energy production (Getino et al., 2024). Nitrogen is vital for synthesizing amino acids, nucleotides, and other cellular constituents. When nitrogen is limited, microorganisms often redirect their metabolic pathways toward accumulating storage compounds like PHAs as a survival strategy (Ma et al., 2024; Rueda et al., 2024). Similarly, phosphorus is a key component of nucleic acids and ATP, and its limitation can trigger PHA accumulation (Korkakaki et al., 2017). Sulfur, required for synthesizing certain amino acids and coenzymes, also influences PHA production, although its role is less pronounced than nitrogen and phosphorus (Tables 2, 3).

Micronutrients, including trace elements like magnesium (Mg), calcium (Ca), iron (Fe), and trace metals such as cobalt (Co), copper (Cu), and zinc (Zn), are critical for the function of various enzymes involved in PHA biosynthesis (Sakarika et al., 2023; Getino et al., 2024). For instance, magnesium is a cofactor for enzymes like PHA synthase, which catalyses hydroxyalkanoate monomers’ polymerization into PHAs (Ronďošová et al., 2022; Getino et al., 2024). Iron is essential for the activity of enzymes involved in the electron transport chain and oxidative phosphorylation processes that provide the energy required for PHA synthesis (Sakarika et al., 2023). The availability of these micronutrients can thus directly affect the efficiency of PHA production (Choi and Lee, 1999). Additionally, the balance between macro and micronutrients is crucial; an excess of one nutrient can lead to the depletion of another, thereby affecting overall microbial growth and PHA accumulation (Choi and Lee, 1999). For example, excess nitrogen can suppress PHA synthesis by promoting cell growth and division, while a balanced limitation can enhance PHA yield (Choi and Lee, 1999).

The type of microorganism used also plays a significant role, as different species have varying nutrient requirements and metabolic capabilities. Cyanobacteria and other photosynthetic bacteria, which can utilize CO_2_ directly, often require specific conditions of light and nutrient availability to optimize PHA production (Higuchi-Takeuchi et al., 2016b; Higuchi-Takeuchi and Numata, 2019; Rueda et al., 2024). Genetic and metabolic engineering approaches have enhanced these microorganisms’ nutrient utilization efficiency and PHA biosynthetic pathways (Madison and Huisman, 1999). Researchers can improve PHA yields even under nutrient-limited conditions by overexpressing key enzymes or knocking out competing pathways (Madison and Huisman, 1999). Environmental factors such as pH, temperature, and light intensity interact with nutrient availability to influence PHA production (González-Rojo et al., 2024). Optimal nutrient uptake and metabolic activity conditions must be maintained to achieve high PHA yields (González-Rojo et al., 2024). Hence, understanding and optimizing these nutrient interactions are essential for improving the efficiency and sustainability of PHA production processes.

## 9 Techno-economic analysis of PHA production from CO_2_

The utilization of CO_2_ as a feedstock for PHA production represents a promising pathway toward sustainable and carbon-negative biopolymer manufacturing. Hydrogenotrophic microorganism, such as *C*. *necator* and its engineered strains, have demonstrated effective CO_2_ fixation under autotrophic growth conditions. Under optimised settings, *C. necator* can achieve biomass yields of approximately 0.5–0.6 g dry cell weight (DCW) per Gram of CO_2_, with PHA accumulation reaching up to 80% of CDW, corresponding to 0.4–0.48 g PHA per g CO_2_ consumed (Troschl et al., 2017b; Khosravi-Darani et al., 2013b). Process configurations utilising CO_2_, H_2_, and O_2_ gas mixtures yield carbon conversion efficiencies (CCE) of 35%–40% on a molar basis, nearing the theoretical maximum of 60% defined by the CBB pathway (Tanaka et al., 2022; Claassens et al., 2016). Volumetric productivities reported for autotrophic gas fermentation systems range from 0.2 to 0.6 g/L/h (Karmann et al., 2017), although intensification beyond 1.0 g/L/h is necessary to achieve competitive economics.

Techno-economic analyses estimate that the cost of producing PHA via direct gas fermentation lies between $4.5–6.0/kg PHA when electrolytic H_2_ is priced at ∼$4/kg (Khosravi-Darani et al., 2013b; Haas et al., 2021). More recent projections suggest that if H_2_ costs are reduced to $2/kg, PHA production costs could decrease to $2.5–3.5/kg (Müller et al., 2023). Capital expenditure (CAPEX) requirements for a 10,000 ton/year plant are typically estimated at $80–120 million, largely driven by the need for specialised pressurised bioreactors and advanced gas management systems (Haas et al., 2021).

Innovations such as MES have emerged to further improve sustainability. In MES systems, electroautotrophic bacteria catalyse CO_2_ reduction at cathodes, producing VFAs like acetate and butyrate, which can subsequently be converted into PHAs (Sciarria et al., 2020). MES-derived VFA to PHA processes demonstrated overall carbon conversion efficiencies of up to 41% (kg C in PHB/kg C as CO_2_) (Alvarez Chavez et al., 2022)​. However, MES-based PHA production remains at an early technology readiness level with capital-intensive setups and relatively low current productivities (30–1330 mmol m^-2^ d^-1^) compared to conventional fermentations.

From an environmental standpoint, life cycle assessment (LCA) studies report net CO_2_ sequestration of approximately 2.2 kg CO_2_ per kg of PHA produced, when considering total system emissions (Narancic et al., 2020). The direct gross CO_2_ fixation, based on stoichiometric calculations, approximates 2.8–3.0 kg CO_2_ per kg of PHA synthesized (Volova et al., 2010). Commercially, Newlight Technologies (United States) has achieved notable advancements, reporting methane/CO_2_-derived AirCarbon^®^ PHA production costs of approximately $2.5–$3.0/kg at pilot scale (Newlight Technologies, 2024). Market projections suggest that reaching PHB production costs below $3/kg and sustaining CO_2_ uptake efficiencies above 0.5 g PHA/g CO_2_ could open access to a global market exceeding 500,000 metric tons annually by 2030, driven by demand in biodegradable packaging, agricultural films, and biomedical applications (European Bioplastics, 2023).

## 10 Challenges and prospects

Harnessing microbial processes to fix CO_2_ offers a compelling eco-friendly approach to mitigating climate change while generating valuable products precisely from CO_2_. Bioconversion of CO_2_ is highly promising because it can lower costs associated with feedstock and operations and simultaneously address energy needs and CO_2_-related environmental risks. However, there are substantial obstacles that must be overcome for successful commercialization. These challenges range from biological constraints and metabolic inefficiencies to economic and engineering difficulties. The primary challenges in autotrophic PHA production using CO_2_ include.

- *Bioprospecting of autotrophic PHA producers*: A key challenge in producing PHAs is identifying robust strains that effectively utilize CO_2_ as a carbon source. Photosynthetic and H_2_-oxidising microorganisms are frequently employed due to their CO_2_ fixation potential. However, achieving commercially viable PHA yields requires selecting naturally proficient strains or applying advanced genetic/metabolic engineering techniques and adaptive evolution strategies to enhance PHA biosynthesis.
- *Slow growth rate*: Autotrophs typically exhibit slower growth rates than heterotrophs, which impacts biomass production and limits PHA yield over time. The extended doubling times observed in cyanobacteria and other autotrophic organisms lead to prolonged production cycles, which pose a significant challenge for large-scale industrial PHA production.
- *Difficulties achieving optimal growth*: Optimizing growth parameters is vital for enhancing PHA production in autotrophic systems. Critical factors like light intensity, CO_2_ concentration, temperature, and nutrient availability significantly influence the efficiency of PHA synthesis. Cyanobacteria require a steady supply of essential micronutrients like nitrogen and phosphorus to maintain growth and PHA synthesis. Managing the availability and recycling of these nutrients at an industrial scale presents logistical challenges, which can negatively impact productivity and increase operational costs. Therefore, fine-tuning these growth conditions is essential for maximizing yield while minimizing resource consumption.
- *CO*_*2*_ *fixation issues*: CO_2_ fixation via autotrophic pathways, mainly through the CBB pathway, is inherently slow, primarily due to the inefficiency of the RuBisCO. This enzyme catalyzes the initial step of the CBB pathway at a sluggish rate. It is prone to oxygenation reactions, which further hinder the conversion of CO_2_ into intermediates for PHA production. Overcoming these limitations necessitates targeted genetic and metabolic engineering approaches to improve RuBisCO’s efficiency. However, these strategies remain in the early stages of development.
- *Metabolic constraints:* In autotrophic organisms, most of the metabolic energy is directed towards carbon fixation, which can limit the resources available for PHA biosynthesis. Consequently, the synthesis of PHAs competes with critical cellular functions, including growth and maintenance. To enhance PHA production, regulating metabolic fluxes is essential to ensure adequate energy and precursor molecules are channelled toward PHA synthesis without compromising cell growth or viability. Achieving this balance is critical to improving the efficiency of PHA production in autotrophic systems.
- *High energy inputs*: Autotrophic PHA production often demands significant energy inputs, mainly light, CO_2_, and essential nutrients. Photoautotrophic systems rely on a constant supply of light energy. Still, the photosynthetic process is relatively inefficient in converting light into chemical energy, limiting these organisms’ overall productivity. Similarly, chemolithotrophs require a steady supply of H_2_ to drive CO_2_ reduction, as CO_2_ is the most oxidized form of carbon and necessitates an external energy source for conversion into more reduced compounds, such as PHAs. As a result, maintaining a continuous flow of these energy resources elevates operational costs, posing challenges for large-scale production.
- *Low PHA yield*: Autotrophic microorganisms generally achieve lower PHA yields than heterotrophic systems. For instance, heterotrophic bacteria can accumulate up to 90% of their DCW as PHA, while autotrophic systems typically demonstrate much lower accumulation levels. Nutrient-limiting conditions such as nitrogen or phosphorus stimulate heterotrophs to store PHAs as energy reserves. In contrast, inducing similar stress conditions in autotrophic systems while ensuring adequate CO_2_ fixation for growth presents a more intricate challenge. Consequently, achieving high PHA yields in autotrophic pathways requires careful management of both stress factors and growth conditions.
- *CO*_*2*_ *availability limitations*: Another major challenge is maintaining a reliable and sufficient supply of CO_2_. Large-scale industrial production demands substantial CO_2_, which can be obtained from industrial emissions or direct air capture sources. However, the associated costs and logistical complexities of CO_2_ capture and transportation can be significant barriers. In addition, maximizing the efficiency of CO_2_ utilization by microorganisms is crucial for ensuring the economic feasibility of the production process.
- *Mass transfer challenges*: Efficient CO_2_ mass transfer in bioreactors is a significant challenge. Unlike soluble carbon sources in heterotrophic PHA production, CO_2_ must be supplied continuously in gaseous form, and its low solubility in water limits availability to microorganisms. Addressing this in large-scale systems requires energy-intensive mixing or solubilization. Maintaining high CO_2_ concentrations while removing O_2_ and other by-products adds complexity to chemolithoautotrophic setups. Additionally, CO_2_ dissolution lowers pH, creating acidic conditions that can inhibit microbial growth and metabolism, thus reducing PHA productivity. Alternatively, pH buffering strategies will be required.
- *Impact from other gases*: Including gases like H_2_, CO, and CH_4_ alongside CO_2_ impacts PHA production. H_2_ supports ATP generation, while CO may inhibit metabolism. CH_4_ provides an alternative carbon source, boosting yields through methanotrophic activity. Hence, optimizing gas composition is crucial for substrate utilization, enhancing productivity, and avoiding metabolic inefficiencies.

Addressing these challenges may require the development of multidisciplinary engineering strategies. This involves integrating bioprocess engineering (gas fermentation), genetic engineering, metabolic engineering, and bioelectrochemistry. Some industries are actively developing biotechnological platforms to convert CO_2_ and CH_4_ into PHAs (Table 6). Although microbial production of PHA from CO_2_ is actively studied, it typically yields lower PHB accumulations than sugar-based methods or even through organic wastes as feedstocks. Several strategies must be developed to improve productivity, enhance CO_2_ utilization, and produce copolymers with excellent physical properties. Exploitation of industrial off-gases is a possible solution to address the CO_2_ availability issue. However, industrial waste gases and syngas usually contain CO, which is toxic to most organisms, making it necessary to engineer CO_2_-utilizing microbes for CO tolerance. Strain development through genetic engineering and metabolic pathway optimization has shown promise and needs further development, particularly for large-scale applications. Additionally, metabolic engineering should focus on producing a range of copolymers. Life cycle assessments are essential to evaluate the CO_2_ mitigation potential and the impact on the circular economy. Simulation modelling of bioprocesses could also play a role in optimizing CO_2_ mitigation and PHA production processes. Moreover, novel online monitoring systems and enhanced process control strategies are necessary for efficient scale-up. Techno-economic analyses should also ensure that CO_2_ conversion into value-added products is cost-effective and sustainable for long-term industrial application.

**TABLE 6:** Companies Producing PHA from CO_2_/CH_4_ gases.

Company	Country	Carbon source	Microbial system	Production strategy	TRL/Scale	PHA products/Applications	References
Kaneka Corporation	Japan	CO_2_ + H_2_	-	Gas fermentation using H_2_ and CO_2_; autotrophic production of PHB/PHBV	Pilot → Semi-commercial	Green Planet™ PHA; biodegradable plastics for packaging, agriculture	Kaneka Corporation (2023)
CO2BioClean GmbH	Germany	Industrial emission CO_2_	-	Proprietary microbial gas fermentation	Pilot	General-use PHA	CO2BioClean (2024)
Bio-on (Lux-on)	Italy	Atmospheric CO_2_+ H_2_ (solar)	-	Solar-driven bioreactor; energy storage with H2; continuous gas fermentation	Research/Pilot	PHA bioplastics for packaging, cosmetics, agriculture	Bio-on and Gruppo Hera pursue development of CO2-based PHA (2018)
Mango Materials	United States	Methane (CH_4_)	Methanotrophs	CH_4_ fermentation to produce PHB; aerobic methanotrophic bioreactor	Pilot/Demo	Biodegradable PHA; textiles, packaging, insulation	Plastics Engineering (2024)
Newlight Technologies	United States	CH_4_, CO_2_	-	AirCarbon^®^ process; large-scale fermentation using biogas	Commercial	AirCarbon^®^ biopolymers (cutlery, fashion, eyewear)	Newlight Technologies (2024); Smith (2023)
Yield10 Bioscience	United States	CO_2_ (photosynthesis)	Engineered *Camelina sativa* (transgenic plant)	PHB biosynthetic genes expressed in oilseed for direct CO_2_ fixation via photosynthesis	Field trials/Lab	PHB from seeds; biodegradable plastics, feed	Yield10 Bioscience (2023)

## 11 Conclusion

This review systematically examines recent advancements in utilizing autotrophic microorganisms for PHA production using CO_2_ as a carbon source. Despite the promising potential, these technologies are still in their infancy and require further expansion to achieve industrial-scale production. Identifying and addressing the critical challenges of the CO_2_-based autotrophic processes through integrated engineering approaches is crucial for advancing autotrophic PHA production platforms. Comprehensive research on improving CO_2_ fixation, PHA biosynthetic pathways, scale-up for process engineering, gas fermentation simulation models, and gas mass transfer could enhance the direct conversion of CO_2_ to PHA by autotrophic microorganisms. Concurrently, ongoing efforts to strengthen promising strains’ adaptability and genetic traits are necessary. These combined efforts will create an eco-friendly process for PHA production using CO_2_ by autotrophic microorganisms.
