# Supplementary material for: Autotrophic bacterial production of polyhydroxyalkanoates using carbon dioxide as a sustainable carbon source
Source: Front Bioeng Biotechnol. 2025 Jun 4;13:1545438. doi: 10.3389/fbioe.2025.1545438 (PMC12174442; doi:10.3389/fbioe.2025.1545438)
Supplement: Supplementary file 1 [file DataSheet1.pdf]

Supplementary Material

Autotrophic Bacterial Production of Polyhydroxyalkanoates Using Carbon Dioxide as a Sustainable Carbon Source

Ganesan Sathiyanarayanan[1*] and Sandra Esteves[1*]

[1]Wales Centre of Excellence for Anaerobic Digestion, Sustainable Environment Research Centre, University of South Wales, Pontypridd, CF37 1DL, Wales, UK

*Contact: sathiyanarayanan.ganesan1@southwales.ac.uk; sandra.esteves@southwales.ac.uk

1 Supplementary Data

Methods for Figure 1

The evolutionary history was inferred using the Neighbor-Joining method (Saitou and Nei, 1987). The optimal tree is shown. The evolutionary distances were computed using the Maximum Composite Likelihood method (Tamura et al., 2004) and are in the units of the number of base substitutions per site. This analysis involved 32 nucleotide sequences. All ambiguous positions were removed for each sequence pair (pairwise deletion option). There were a total of 1623 positions in the final dataset. Evolutionary analyses were conducted in MEGA11 (Stecher et al., 2020; Tamura et al., 2021)

Methods for Figure 2

The evolutionary history was inferred using the Neighbor-Joining method (Saitou and Nei, 1987). The optimal tree is shown. The evolutionary distances were computed using the Maximum Composite Likelihood method (Tamura et al., 2004) and are in the units of the number of base substitutions per site. This analysis involved 68 nucleotide sequences. All ambiguous positions were removed for each sequence pair (pairwise deletion option). There were a total of 2128 positions in the final dataset. Evolutionary analyses were conducted in MEGA11 (Stecher et al., 2020; Tamura et al., 2021)

2 Supplementary Tables

Table S1. Photo-mixotrophic production of PHA from cyanobacteria

Cyanobacterial strain	Carbon source	PHA content in DCW (%)	Limiting factor	PHA composition	Bioreactor	Reference
Arthrospira platensis UMACC 161	CO_2 (0.04%) + 0.5% Acetate	10	N	P (3HB)	Erlenmeyer flask	(Toh et al., 2008)
Chlorogloea fritschii	CO_2 (5%) + 0.02M acetate	10	-	PHB	Erlenmeyer flask	(Carr, 1966)
Chlorogloeopsis fritschii PCC 6912	CO_2 (0.04%) + 15 mM Acetate	6.2	N	PHB	Erlenmeyer flask	(Hai et al., 2001)
Cyanothece sp. PCC 7424	CO_2 (0.04%) + 15 mM Acetate	1.1	N	PHB	Erlenmeyer flask	(Hai et al., 2001)
Cyanothece sp. PCC 8303	CO_2 (0.04%) + 15 mM Acetate	2.7	N	PHB	Erlenmeyer flask	(Hai et al., 2001)
Desmonostoc muscorum SAG 1453-12b (*Nostoc muscorum* Agardh)	CO_2 (10%)[a]	70	-	PHB-co-PHV	CSTR	(Bhati and Mallick, 2016)
Desmonostoc muscorum SAG 1453-12b (*Nostoc muscorum* Agardh)	CO_2 (10%)[a]	65	-	PHB-co-PHV	CSTR	(Bhati and Mallick, 2016)
Gloeocapsa sp. PCC 7428	CO_2 (0.04%) + 15 mM Acetate	1.5	N	PHB	Erlenmeyer flask	(Hai et al., 2001)
Gloeothece sp. PCC 6501	CO_2 (0.04%) + 15 mM Acetate	1.4	N	PHB	Erlenmeyer flask	(Hai et al., 2001)
Gloeothece sp. PCC 6909	CO_2 (0.04%) + 10 mM Acetate	6-9	-	PHB	Erlenmeyer flask	(Stal, 1992)
Stanieria sp. PCC 7437	CO_2 (0.04%) + 15 mM Acetate	0.9	N	PHB	Erlenmeyer flask	(Hai et al., 2001)
Synechococcus elongatus	CO_2 (0.04%) + 1% Sucrose	17.15	N	PHA[b]	Erlenmeyer flask	(Mendhulkar and Shetye, 2017)

Synechococcus elongatus	CO_2 (0.04%) + 1% Fructose	7.02	P	PHA[b]	Erlenmeyer flask	(Mendhulkar and Shetye, 2017)
Synechococcus sp. MA19	CO_2 (0.04%) + 15 mM Acetate	6.5	N	PHB	Erlenmeyer flask	(Hai et al., 2001)
Synechocystis PCC 6803	CO_2 (0.04%) + 0.4% acetate	44–48	N	PHB	Erlenmeyer flask	(Dutt and Srivastava, 2018)
Synechocystis sp. UNIWG	CO_2 (0.04%) + 0.5% Acetate	14	N	PHB	Erlenmeyer flask	(Toh et al., 2008)

[a] Optimized substrates: 0.28 % acetate, 0.38 % glucose, and 0.30 % valerate.

[b] not mentioned

Table S2. Mixotrophic production of PHA from *C. necator*.

Microorganism	Substrate used	Process type	Biopolymer Yield (g/L or % in DCW)	Limiting factor	PHA composition	Bioreactor	References
C. necator B-10646	CO_2:O_2:H_2 (1:2:7) + Propionate, valerate, hexanoate, c-butyrolactone	Batch, Fed-batch	42.5 g/L (85%)	N	PHB	CSTR	(Volova et al., 2013)
			-	N, K, P, S, Mn	4HB, 3HV, 3HHx, 3HO	CSTR	
C. necator DSM 545	H_2:O_2:CO_2 (84:2.8:13.2 vol%) + Valerate	Fed-batch, semi-continuous	24.7 g/L (78%)	Valerate	PHB-co-PHV	CSTR	(Ghysels et al., 2018)
C. necator ATCC 17697	CO_2:H_2:O_2:N_2 (1:7:1: 91%) + Valerate	Batch, Fed-batch	1.07 g/L	N	PHB-co-PHV	Erlenmeyer flask	(Park et al., 2014)
C. necator B-5786	CO_2:O_2:H_2 (1:2:6) + Valerate	Two-stage batch	8-10 g/L (60%)	Valerate	PHB-co-PHV	CSTR	(Volova and Kalacheva, 2005)
C. necator B-5786	CO_2:O_2:H_2 (1:2:6)	Batch	12 g/L (63%)	N	PHB	CSTR	(Volova et al., 2004)
C. necator DSM 545	H_2:O_2:CO_2 (84:2.8:13.2 vol%) + Glycerol	Fed-batch	28 g/L	N, O_2	PHB	CSTR	(Garcia-Gonzalez et al., 2015)
C. necator H16 and B5786	CO_2	Fed-batch	63.0 & 61.4 %	N	PHB-co-PHV-co-PHHx	Erlenmeyer flask	(Volova et al., 2008)

C. necator ATCC 17697[T]	H_2:O_2:CO_2 (75:15:10)	Batch	36 g/L	H_2, O_2, N	PHB	CSTR	(Ishizaki and Tanaka, 1991)
C. necator DSM 545	H_2:O_2:CO_2 (84:2.8:13.2) vol%	Two-phase batch	24 g/L (63%)	N	PHB	CSTR	(Garcia-Gonzalez and De Wever, 2017)

References:

Bhati, R., and Mallick, N. (2016). Carbon dioxide and poultry waste utilization for production of polyhydroxyalkanoate biopolymers by *Nostoc muscorum* Agardh: a sustainable approach. *J Appl Phycol* 28, 161–168. doi:10.1007/s10811-015-0573-x

Carr, N. G. (1966). The occurrence of poly-β-hydroxybutyrate in the blue-green alga, *Chlorogloea fritschii*. *Biochimica et Biophysica Acta (BBA) - Biophysics including Photosynthesis* 120, 308–310. doi:10.1016/0926-6585(66)90353-0

Dutt, V., and Srivastava, S. (2018). Novel quantitative insights into carbon sources for synthesis of poly hydroxybutyrate in *Synechocystis* PCC 6803. *Photosynth Res* 136, 303–314. doi:10.1007/s11120-017-0464-x

Garcia-Gonzalez, L., and De Wever, H. (2017). Valorisation of CO_2-rich off-gases to biopolymers through biotechnological process. *FEMS Microbiol Lett* 364, fnx196. doi:10.1093/femsle/fnx196

Garcia-Gonzalez, L., Mozumder, Md. S. I., Dubreuil, M., Volcke, E. I. P., and De Wever, H. (2015). Sustainable autotrophic production of polyhydroxybutyrate (PHB) from CO_2 using a two-stage cultivation system. *Catal Today* 257, 237–245. doi:10.1016/j.cattod.2014.05.025

Ghysels, S., Mozumder, Md. S. I., De Wever, H., Volcke, E. I. P., and Garcia-Gonzalez, L. (2018). Targeted poly(3-hydroxybutyrate-co-3-hydroxyvalerate) bioplastic production from carbon dioxide. *Bioresour Technol* 249, 858–868. doi:10.1016/j.biortech.2017.10.081

Hai, T., Hein, S., and Steinbüchel, A. (2001). Multiple evidence for widespread and general occurrence of type-III PHA synthases in cyanobacteria and molecular characterization of the PHA synthases from two thermophilic cyanobacteria: *Chlorogloeopsis fritschii* PCC 6912 and *Synechococcus* sp. strain MA19. *Microbiology (N Y)* 147, 3047–3060. doi:10.1099/00221287-147-11-3047

Ishizaki, A., and Tanaka, K. (1991). Production of poly-β-hydroxybutyric acid from carbon dioxide by *Alcaligenes eutrophus* ATCC 17697^T. *J Ferment Bioeng* 71, 254–257. doi:10.1016/0922-338X(91)90277-N

Mendhulkar, V. D., and Shetye, L. A. (2017). Synthesis of Biodegradable Polymer Polyhydroxyalkanoate (PHA) in Cyanobacteria *Synechococcus elongates* Under Mixotrophic Nitrogen- and Phosphate-Mediated Stress Conditions. *Industrial Biotechnology* 13, 85–93. doi:10.1089/ind.2016.0021

Park, I., Jho, E. H., and Nam, K. (2014). Optimization of Carbon Dioxide and Valeric Acid Utilization for Polyhydroxyalkanoates Synthesis by *Cupriavidus necator*. *J Polym Environ* 22, 244–251. doi:10.1007/s10924-013-0627-6

Saitou, N., and Nei, M. (1987). The neighbor-joining method: a new method for reconstructing phylogenetic trees. *Mol Biol Evol* 4, 406–425. doi:10.1093/oxfordjournals.molbev.a040454

Stal, L. J. (1992). Poly(hydroxyalkanoate) in cyanobacteria: an overview. *FEMS Microbiol Lett* 103, 169–180. doi:10.1016/0378-1097(92)90307-A

Stecher, G., Tamura, K., and Kumar, S. (2020). Molecular Evolutionary Genetics Analysis (MEGA) for macOS. *Mol Biol Evol* 37, 1237–1239. doi:10.1093/molbev/msz312

Tamura, K., Nei, M., and Kumar, S. (2004). Prospects for inferring very large phylogenies by using the neighbor-joining method. *Proceedings of the National Academy of Sciences* 101, 11030–11035. doi:10.1073/pnas.0404206101

Tamura, K., Stecher, G., and Kumar, S. (2021). MEGA11: Molecular Evolutionary Genetics Analysis Version 11. *Mol Biol Evol* 38, 3022–3027. doi:10.1093/molbev/msab120

Toh, P., Jau, M. H., Yew, S.-P., Abed, R., and Sudesh, K. (2008). Comparison of polyhydroxyalkonates biosynthesis, mobilization and the effects of cellular morphology in *Spirulina platensis* and *Synechocystis* sp. UNIWG. J Biosci. 19, 21–38.

Volova, T. G., and Kalacheva, G. S. (2005). The synthesis of hydroxybutyrate and hydroxyvalerate copolymers by the bacterium *Ralstonia eutropha*. *Microbiology (N Y)* 74, 54–59. doi:10.1007/s11021-005-0028-5

Volova, T. G., Kalacheva, G. S., Gorbunova, O. V, and Zhila, N. O. (2004). Dynamics of Activity of the Key Enzymes of Polyhydroxyalkanoate Metabolism in *Ralstonia eutropha* B5786. *Appl Biochem Microbiol* 40, 170–177. doi:10.1023/B:ABIM.0000018921.04863.d5

Volova, T. G., Kalacheva, G. S., and Steinbüchel, A. (2008). Biosynthesis of Multi-Component Polyhydroxyalkanoates by the Bacterium *Wautersia eutropha*. *Macromol Symp* 269, 1–7. doi:10.1002/masy.200850901

Volova, T. G., Kiselev, E. G., Shishatskaya, E. I., Zhila, N. O., Boyandin, A. N., Syrvacheva, D. A., et al. (2013). Cell growth and accumulation of polyhydroxyalkanoates from CO_2 and H_2 of a hydrogen-oxidizing bacterium, *Cupriavidus eutrophus* B-10646. *Bioresour Technol* 146, 215–222. doi:10.1016/j.biortech.2013.07.070
